# Reliable Apparent Permeability Prediction Through Benchmarking, Stability, Uncertainty Quantification, and Explainable Machine Learning

**DOI:** 10.1002/psp4.70305

**Published:** 2026-08-13

**Authors:** Rajdeep Mondal, Rajith K. R. Rajoli, Andrew Owen, Soumitra Samanta

**Affiliations:** ^1^ Ramakrishna Mission Vivekananda Educational and Research Institute Belur India; ^2^ Institute for Advancing Intelligence, TCG CREST Kolkata India; ^3^ Centre of Excellence for Long‐Acting Therapeutics, University of Liverpool Liverpool UK; ^4^ Department of Pharmacology and Therapeutics University of Liverpool Liverpool UK

**Keywords:** apparent permeability, interpretable QSAR modeling, molecular representation learning, multi‐relational graph neural networks, uncertainty quantification

## Abstract

Apparent permeability ($P_{\mathrm{app}}$) of a drug molecule serves as a principal indicator of drug absorption for pharmacokinetic modeling, and accurate prediction of $P_{\mathrm{app}}$ values using machine learning would avoid time‐consuming and expensive In Vitro experiments. In this study, four different feature representations were compared for predicting $\log P_{\mathrm{app}}$ values, with each representation serving as input to three subsequent regression models. The best predictive model achieved an $R^{2}$ value of 0.68 and RMSE of 0.42 on the test data. Furthermore, the SHAP values corresponding to individual molecular descriptors were analyzed to interpret the influence of these descriptors on the model prediction. Some key properties like octanol–water partition coefficient and number of basic atoms were found to have a strong influence on the $P_{\mathrm{app}}$ value. Additionally, some specific molecular substructures were identified that contribute either positively or negatively to the model output, thereby inferring effects on drug permeability. Finally, the uncertainty in the predictions was quantified through a distribution‐free and model‐agnostic method, *jackknife+*, used for the estimation of confidence intervals. At the optimal experimental setting, the confidence intervals derived using the overall best performing model covered 96.12% and 86.73% of the original $\log P_{\mathrm{app}}$ values with more than 95% confidence for test and independent data, respectively. With the same confidence, the confidence intervals contained 99.87% and 100% of the predicted $\log P_{\mathrm{app}}$ values for the test and independent data, respectively, underscoring the reliability of the model. Overall, the presented ML provides the most robust informed $P_{\mathrm{app}}$ prediction among compared models presented to date.

## Study Highlights

- What is the current knowledge on the topic?
  ○ Machine learning (ML) models have become a cost‐effective strategy for early prediction of a compound's apparent permeability (𝑃𝑎𝑝𝑝). Although numerous ML and deep learning frameworks have been proposed for 𝑃𝑎𝑝𝑝 prediction, they largely function as black‐box models, offering limited interpretability and explainability.
- What question did this study address?
  ○ This study investigated whether the patterns learned by the ML model were consistent with established physicochemical principles by comparing different feature representations of chemical compounds. It also examined uncertainty quantification to assess the reliability of the model.
- What does this study add to our knowledge?
  ○ The study demonstrates that feature representations derived from atom‐level descriptors are more generalizable than those based on molecular‐level descriptors.
  ○ Combining molecular‐level descriptors with Random Forest or neural network models, together with SHAP‐based interpretation, provides a reliable approach for identifying descriptor influence on compound permeability.
  ○ The graph‐based ML model (MRGCN) exhibited stable performance, which was further supported by uncertainty quantification.
- How might this change drug discovery, development, and/or therapeutics?
  ○ Prediction of 𝑃𝑎𝑝𝑝 using ML can accelerate drug discovery by rapidly screening compounds for absorption potential, reducing experimental costs, and guiding medicinal chemistry. It improves early ADME optimization, derisks development and supports better formulation design, ultimately shortening timelines and increasing success rates.

## 1 Introduction

Oral dosing represents the most common route of drug administration. In pharmacokinetics, five fundamental properties, namely absorption, distribution, metabolism, excretion, and toxicity (ADMET), are crucial for understanding the efficacy and safety of a drug to avoid late‐stage clinical failure. Poor drug absorption can lead to attrition even though a drug has good potential to modulate its target. The major regions for drug absorption of orally administered drugs include the stomach, small intestine, and a small part of large intestine [1]. However, as the in vivo determination of drug absorption is not feasible before the preclinical or clinical stages, in vitro techniques play an important role for early identification of low permeable drugs. Norinder et al. reported that the intestinal permeability of a compound correlates with its intestinal absorption and therefore, a drug's absorption in the intestine can be inferred from its permeability [2]. Among several cell cultures used as in vitro surrogates of the human intestine epithelial barrier, Caco‐2 cells, derived from human colorectal adenocarcinoma cells, are most commonly used for in vitro drug permeability assessment [3]. The main limitation of this method is the lengthy culture period of 21–24 days to enable cell differentiation into a robust monolayer [4].

A quantitative structure–property relationship (QSPR) model is a fast, simple and cost‐efficient alternative for in vitro methods [5, 6]. The growing success and widespread applicability of machine learning (ML) techniques across diverse fields enable researchers to leverage these techniques to develop efficient QSPR models [7]. Several classification and regression models using classical machine learning techniques have been proposed to predict the Caco‐2 cell permeability value on relatively small datasets [4, 8, 9, 10]. Wang et al. applied multiple linear regression (MLR), partial least‐square (PLS), support vector machine (SVM), and boosting methods on a small dataset to predict the Caco‐2 permeability value [8]. Chen et al. worked on a relatively larger dataset using dual radial basis function (dual‐RBF) network [9]. A conditional consensus model, made of individual regression random forests has also been proposed by Falcón‐Cano et al. [4] with recursive variable selection technique. Furthermore, Walter et al. demonstrated that multi‐task graph‐based approaches are more effective than single‐task models for ADME predictions if shared information between multiple endpoints can be leveraged together [11]. Some deep learning‐based models like Long Short‐Term Memory (LSTM) and Graph Convolution Network (GCN) have also been reported [10] in the literature.

High experimental variability in the apparent permeability values for the same compound across different datasets is a major challenge to build a ML model for P_app_ value prediction. Moreover, overlapping datapoints are often found in both the train and test data partitions after applying the canonicalization process on the simplified molecular input line entry system (SMILES) string representation of a compound in the source datasets [12]. These overlapping datapoints might lead to inappropriate evaluation of ML models which further results in misleading interpretation of the robustness and fairness of the resulting models. The efficiency and unbiasedness of an ML model rely on the quality of the available data, and to build an efficient model, a clean and reliable dataset is required. Besides, although the representational effectiveness between GCN and molecular descriptor has been compared by Walter et al. a comprehensive comparison across multiple representations‐regressor combinations and more detailed analysis of descriptor‐level contribution quantification remain unexplored for Caco‐2 permeability value prediction.

In this study, four different numerical representations of a compound were explored to represent a SMILES string into a fixed dimensional feature vector. Three popular ML models were then used to build regression models using each of these representations on a dataset curated from three different sources. Additionally, the influence of molecular features on a prediction was also analyzed to interpret the model's reliability through SHAP value analysis of the descriptors [13]. The interpretability analysis was further extended to examine whether any common fragments or substructures have a positive or negative effect on permeability prediction. In this context, positive/negative effect implies an increase/decrease to the prediction value in the presence of a particular substructure within each compound. Finally, a statistical confidence interval estimation method, jackknife+, was implemented to quantify the uncertainty in the model prediction. Comparisons indicated that both the confidence interval estimation and model predictions were efficient and reliable.

## 2 Materials and Methods

### 2.1 Data Collection and Pre‐Processing

The Caco‐2 permeability dataset was constructed from three publicly available sources [8, 9, 10], containing experimentally measured Caco‐2 permeability values for chemical compounds with their corresponding SMILES representation. Additional details on individual source datasets are provided in Data S1.

The datasets were subsequently unified and partitioned into train, validation and test sets as follows: Let $D_{i}$ denote the $i^{\mathrm{th}}$ source dataset, with $D_{i}^{\text{train}}$ and $D_{i}^{\text{test}}$ representing its corresponding train and test subsets, respectively $\left(i=1,2,3\right)$. First, the SMILES from $\overset{3}{\underset{i=1}{⋃}}D_{i}$ were canonicalized using the RDKit library [14] to identify the unique SMILES. The compounds with multiple reported permeability values were assigned the target label as the mean across the reported values. The test partition $\left(D^{\text{test}}\right)$ were first separated from the unified dataset as $\overset{3}{\underset{i=1}{⋃}}D_{i}^{\text{test}}$. From the remaining dataset, the validation set $\left(D^{\mathrm{val}}\right)$ was constituted by randomly selecting 10% datapoints from $D\backslash D^{\text{test}}$, as the source datasets did not provide any explicit validation partition for hyperparameter optimization. Finally the train set was derived as $D^{\text{train}}=D\backslash \left(D^{\text{test}}\cup D^{\mathrm{val}}\right)$.

On the train, validation and test subsets, the following filtering criteria were applied to enrich the datasets: (i) compounds containing only biologically relevant atoms (C, H, N, O, S, P, Br, Cl, F, and I) were retained; (ii) compounds with a molecular weight more than 900 Da were excluded; (iii) compounds with a standard deviation of multiple reported values of $\log P_{\mathrm{app}}$ greater than 0.5 were removed.

During preprocessing, 71, 38, and 40 overlapping data points were identified between the train and test partitions for the source datasets reported in [8, 9, 10], respectively. Overlapping instances can unnecessarily inflate the evaluation metrics of a model, which in turn provides misleading assessment about the generalizability. To address this, mutual exclusiveness between the final train, validation, and test sets was ensured while constructing the data subsets. Additionally, an independent dataset of 182 marketed drugs was also used to evaluate the regression model's effectiveness on real‐world and unseen compounds. The final independent dataset was reduced to 98, after eliminating compounds from the external dataset based on the defined filtering criteria or overlapping datapoints between the train and validation sets. The final data partition contained 3166 train instances, 748 test instances, 349 validation instances and 98 independent instances.

### 2.2 Feature Representation

Four different feature representations of chemical compounds were investigated in this study to assess their effectiveness in predicting the apparent Caco‐2 permeability values. These included a classical molecular descriptor‐based representation, along with three learned representations derived from popular deep learning models: Multi‐relational graph convolutional network (MRGCN) [15], variational auto‐encoder (VAE) [16, 17], and Mol2Vec [18].

#### 2.2.1 Molecular Descriptor Representations

A vector comprising all molecular‐level descriptors, computed using the cheminformatics toolkits—RDKit [14] and Mordred [19], served as the molecular representation of each compound. In total, 2309 descriptors were computed and subsequently processed through a series of feature selection steps to obtain a refined set of 642 molecular descriptors after eliminating non‐informative or redundant features. The feature selection procedure and feature list are detailed in Data S2.1.

#### 2.2.2 Multi‐Relational Graph Convolution Network (MRGCN) Representation

A molecule can be represented as a multi‐relational graph, where atoms correspond to nodes and bonds correspond to edges. Different relationships between the atoms were defined by different bond types (single, double, triple, and aromatic) that present between them. The 3D adjacency tensor and degree tensor implicitly encoded the molecular structures and different relationships, whereas the initial node features containing 17 atom‐level descriptors incorporated the atomic level information of a compound. A multi‐relational graph convolutional network (MRGCN) was employed to obtain a 500‐dimensional numerical representation of a molecule from its adjacency tensor, degree tensor, and node features [20]. More details about the node feature selection, adjacency and degree tensor construction, and MRGCN architecture can be found in Data S2.2.

#### 2.2.3 Variational Auto‐Encoder (VAE) Representation

A variational auto‐encoder model [16], pretrained on approximately 39 million SMILES strings from ZINC dataset with SMILES reconstruction task, was employed to derive a 300‐dimensional continuous representation for each compound. The VAE architecture used to derive the feature representation was similar to the network designed by Samanta et al. [17]. Each SMILES string was encoded as a one‐hot representation based on a predefined vocabulary containing 63 tokens, yielding an input matrix of size 250×63 for the auto‐encoder. The latent dimension of the VAE model was empirically set to 300, and the resulting continuous vector of the same dimension was served as the molecular representation for VAE. More details regarding the VAE model, network architecture, tokenization scheme, and training setup are provided in Data S2.3.

#### 2.2.4 Mol2Vec Representation

Pre‐trained Mol2Vec model provided by Jaeger et al. was used to generate a 300 dimensional continuous vector representation for a given SMILES [18]. The Mol2Vec model treats a molecule as a sentence, and its substructures derived from the Morgan algorithm as words. The numerical representation is obtained by aggregating all the embeddings of the substructures present in the molecule. More details about Mol2Vec model are provided in Data S2.4.

### 2.3 Regression Models

Three regression models were used to predict the permeability values using the four molecular representations described in Section 2.2, namely: neural network (NN), random forest (RF), and epsilon‐support vector regressor (ϵ‐SVR) (outlined in Data S3). This resulted in a total of 12 representation–regressor combinations. Optimal hyperparameters configuration for these combinations is summarized in Tables S4, S5 and S6 for NN, RF and ϵ‐SVR, respectively and the selection strategy is detailed in Data S4. The RF and ϵ‐SVR models were implemented using the Scikit‐learn library [21], while the NN model was designed using the PyTorch framework [22]. The results are reported in Table 1 as mean±std, obtained from five runs with different random seed initializations.

**TABLE 1:** Performance of each regressor model across all the representations, obtained by averaging the results over five independent experiments with different random seed initializations.

Representation	Regressor	$R^{2}$	RMSE	MAE	10%	20%
Test dataset						
Descriptors	𝛜‐SVR	0.68 ± 0.00	0.42 ± 0.00	0.32 ± 0.00	81.42 ± 0.00	99.47 ± 0.00
	RF	0.64 ± 0.02	0.44 ± 0.01	0.35 ± 0.01	76.34 ± 0.69	98.82 ± 0.30
	NN	0.65 ± 0.01	0.43 ± 0.01	0.32 ± 0.01	79.76 ± 0.70	98.48 ± 0.38
MRGCN	𝛜‐SVR	0.65 ± 0.01	0.44 ± 0.01	0.32 ± 0.01	81.56 ± 0.86	97.13 ± 0.45
	RF	0.65 ± 0.01	0.43 ± 0.01	0.31 ± 0.01	82.78 ± 0.95	99.23 ± 0.34
	NN	0.64 ± 0.01	0.45 ± 0.01	0.34 ± 0.01	80.32 ± 1.31	97.51 ± 0.35
Mol2Vec	𝛜‐SVR	0.57 ± 0.00	0.48 ± 0.00	0.37 ± 0.00	74.87 ± 0.00	98.40 ± 0.00
	RF	0.59 ± 0.03	0.48 ± 0.02	0.38 ± 0.02	73.40 ± 0.56	98.13 ± 0.41
	NN	0.57 ± 0.01	0.48 ± 0.01	0.35 ± 0.02	76.87 ± 0.43	96.55 ± 0.67
VAE	𝛜‐SVR	0.45 ± 0.00	0.55 ± 0.00	0.42 ± 0.00	69.25 ± 0.00	95.05 ± 0.00
	RF	0.40 ± 0.01	0.57 ± 0.02	0.45 ± 0.01	64.04 ± 2.59	95.45 ± 0.98
	NN	0.41 ± 0.01	0.57 ± 0.01	0.43 ± 0.01	69.25 ± 0.55	94.79 ± 1.21
Independent dataset						
Descriptors	𝛜‐SVR	0.44 ± 0.00	0.64 ± 0.00	0.48 ± 0.00	69.39 ± 0.00	90.82 ± 0.00
	RF	0.45 ± 0.02	0.63 ± 0.02	0.45 ± 0.01	65.31 ± 0.00	92.86 ± 0.21
	NN	0.45 ± 0.01	0.63 ± 0.01	0.46 ± 0.01	67.96 ± 1.22	90.82 ± 0.65
MRGCN	𝛜‐SVR	0.50 ± 0.02	0.60 ± 0.01	0.44 ± 0.01	70.55 ± 1.34	92.74 ± 1.59
	RF	0.54 ± 0.02	0.58 ± 0.02	0.42 ± 0.02	72.65 ± 1.63	93.04 ± 1.19
	NN	0.49 ± 0.02	0.61 ± 0.01	0.46 ± 0.02	68.37 ± 1.44	91.64 ± 1.98
Mol2Vec	𝛜‐SVR	0.36 ± 0.00	0.69 ± 0.00	0.52 ± 0.00	61.22 ± 0.00	88.78 ± 0.00
	RF	0.34 ± 0.01	0.69 ± 0.01	0.56 ± 0.01	60.20 ± 1.36	90.82 ± 0.29
	NN	0.43 ± 0.03	0.65 ± 0.02	0.49 ± 0.02	64.08 ± 1.64	89.80 ± 0.91
VAE	𝛜‐SVR	0.10 ± 0.00	0.81 ± 0.00	0.64 ± 0.00	48.98 ± 0.00	82.95 ± 0.00
	RF	0.17 ± 0.01	0.78 ± 0.01	0.63 ± 0.01	44.90 ± 0.98	85.71 ± 1.07
	NN	0.11 ± 0.01	0.81 ± 0.01	0.64 ± 0.01	52.04 ± 1.02	83.67 ± 1.93

*Note:* $R^{2}$—Coefficient of determination, RMSE—root mean squared error & MAE—mean absolute error value between the original and predicted $\log P_{\mathrm{app}}$ value. 10%, 20%: Percentage of datapoints having prediction error less than 10% and 20%, respectively, with respect to the original $\log P_{\mathrm{app}}$ value.

### 2.4 Evaluation Metrics

Three widely recognized metrics, namely: the coefficient of determination ($R^{2}$), root mean squared error (RMSE) and mean absolute error (MAE) were adopted to evaluate the model performances. Higher $R^{2}$ value, lower RMSE and MAE values represent an improvement in predictive performance. In addition, 10% and 20% error margins were also computed to evaluate the model performances. In case of the best performing model, Bland–Altman (BA) plots were also constructed for both the test and independent sets using one instance of the model to further assess the agreement between true and predicted values (details are in the Data S5).

Furthermore, to ensure whether the data partitioning used in this study was unbiased toward any particular split, 10‐fold cross validation was employed across all the representation‐regressor combinations. In addition, Bemis‐Murcko scaffold‐based splitting [23] was applied on the dataset to generate structurally dissimilar data partitions. The out‐of‐distribution (OOD) generalizability of the investigated combinations was assessed using these scaffold‐based partitions, as detailed in Data S6.

### 2.5 Model Interpretability

The feature influence quantifies the contribution of individual features in predicting the $\log P_{\mathrm{app}}$ value for a given compound. Feature importance was based on the mean absolute Shapley additive explanations (SHAP) values [13] for molecular descriptors and identified the most prominent contributors. For the other three feature representations (MRGCN, VAE, and Mol2Vec), explicit physicochemical interpretability is not feasible, as these embeddings reside in low‐dimensional latent spaces.

Among regressors, we focused on RF and NN models, since calculating SHAP values for ϵ‐SVR with an RBF kernel is computationally expensive and challenging [24]. For the RF model, we additionally computed feature importance using the Gini‐index [25], which allowed us to cross‐validate the predictive relevance of the top ranked features. For NN models, no such direct method exists, making SHAP a valuable tool for feature attribution.

Furthermore, the uncertainty behavior of all the representation‐regressor combinations was assessed using the *jackknife +* framework for confidence interval estimation, detailed in Data S7. In addition, the root mean square error (RMSE) and root mean variance (RMV) across all the 10‐fold results were compared to identify the representation exhibiting the most favorable uncertainty behavior (experimental details and results are in Data S8). Finally, we analyzed the most common influential molecular substructures for both NN and RF models using 1024‐bit Morgan fingerprints of radius 2 [14]. This substructure‐level interpretation provided insights into whether specific functional groups or scaffolds tend to exert a positive or negative influence on $\log P_{\mathrm{app}}$. In SHAP interpretation, a positive SHAP value implies that the feature has a positive impact on the model output and vice versa.

## 3 Results

Table 1 presents comparative results for all the representation‐regressor combinations on both the test and independent test set. Molecular descriptors representation with the ϵ‐SVR model achieved the highest $R^{2}$ value of 0.68 on the test set. However, in terms of MAE, the best predictive performance was obtained by the MRGCN representation with the RF model (MAE=0.31). On the independent test set, the performance of the molecular descriptor–based representation dropped ($R^{2}=0.44$, MAE=0.48), whereas the MRGCN representation with RF achieved the best results ($R^{2}=0.54$, MAE=0.42). Although the improvement over the molecular descriptor‐based models was modest with respect to MAE, gain for $R^{2}$ value was notable. For the Mol2Vec‐based representation, the RF model achieved a strong correlation with an $R^{2}$ value of 0.59 and MAE of 0.38 on the test set.

**FIGURE 1:**
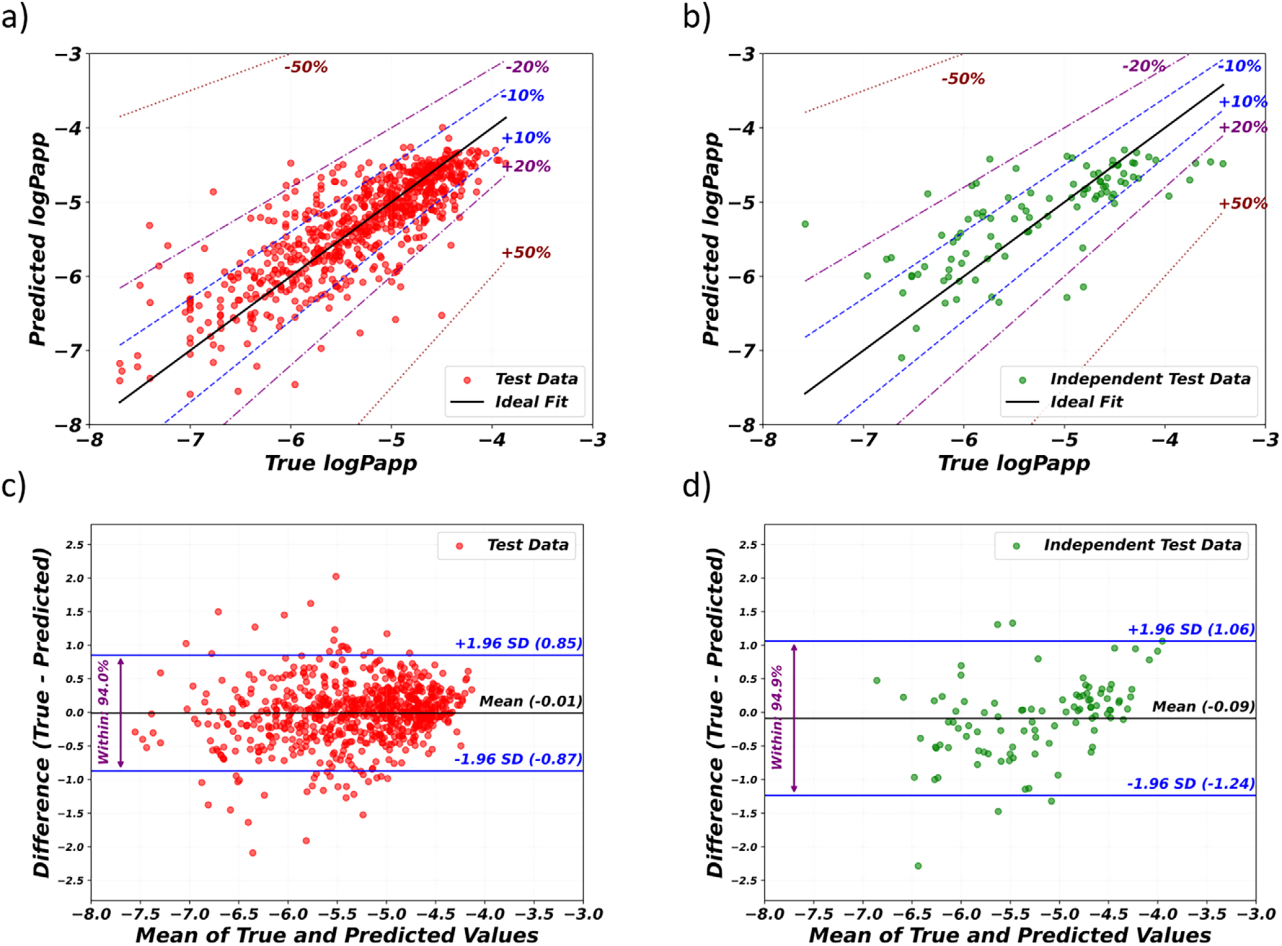
MRGCN with RF model performance plot on test and independent test sets: (a) and (b) Original vs. predicted $\log P_{\mathrm{app}}$ values with percentage (%) errors on test and independent test sets respectively. The dashed blue lines on the both sides of solid black line (i.e., the ideal fit line y=x) indicated the interval $\left[t-\frac{10t}{100},t+\frac{10t}{100}\right]$ corresponding to the 10% error margin, where t is the original value indicated by the X‐axis. Similarly, the violet dashed dot line and the purple dotted line correspond to the 20% and 50% error margins, respectively. (c) and (d) Bland–Altman plots between ground‐truth and predicted $\log P_{\mathrm{app}}$ values on test and independent test sets, respectively. The solid blue line in figures (c) and (d) indicates the 95% limits of agreement defined as $\left[\text{mean}-1.96\times \mathrm{SD},\text{mean}+1.96\times \mathrm{SD}\right]$ and the solid black line represents the mean bias. SD: Standard deviation. In all these figures, red and green color correspond to the test and independent test instances, respectively.

Table 1 also reports the 10% and 20% error margins for all representation‐regressor combinations across both test sets. The MRGCN with RF model achieved the best performance in almost all cases. Figure 1a,b illustrate the predictions for the MRGCN with RF model with 10%, 20%, and 50% error margins plotted around the ideal fit line. From these results, it is evident that the majority of predictions fall within the 20% error margin, highlighting the strong predictive ability of the model across varying error thresholds. In addition, 2D‐visualization of the molecular embeddings are depicted in Figure S2.

Figure 1c,d present the BA plots for MRGCN with RF model on both the test and independent datasets. The near‐zero systematic bias for both the test (−0.01) and independent (−0.09) datasets suggested that the model did not consistently overestimate or underestimate the predicted values. Notably, 94% and 94.9% of the differences fell within the limits of agreement for both the test and independent sets, respectively, highlighting the reliability of the model's predictions across different datasets. Interestingly, when a reference threshold value of −5.5 was set for the *mean* $\log P_{\mathrm{app}}$ values in both datasets, it was observed that the prediction error was generally lower for compounds with $\log P_{\mathrm{app}}\geq -5.5$ compared to those with $\log P_{\mathrm{app}}\leq -5.5$. This indicates that the model performs more accurately for molecules with higher permeability values than those with lower permeability values.

The 10‐fold cross validation results from Table S8 demonstrated that the achieved performance is unbiased toward any specific partitions. In addition, as evidenced from Table S9, the MRGCN + RF model achieved superior performance among all the investigated combinations with $R^{2}$ value of 0.69 and 0.56, RMSE of 0.42 and 0.57, MAE of 0.32 and 0.38 on the test and independent dataset, respectively. Although the performance gap with its closest competing combinations is small, this represents another instance where the MRGCN+RF model demonstrates better performance. Results summarized in Tables S8 and S9 also indicated that the models exhibited robust and stable performance across all configurations and different data partitioning.

### 3.1 Uncertainty Measurement of the MRGCN With RF Model

Table 2 presents all the results obtained from the uncertainty quantification for the best‐performing model (MRGCN with RF) using the jackknife+ method [26]. A brief description about jackknife+, and results for remaining representation‐regressor combinations are provided in Data S7. For α=0.001,0.005, the confidence values were high (97.41% and 96.61%), but the CIs were wide (test: 3.30,2.10; independent: 3.40,2.10), indicating inefficiency. Conversely, for α=0.05,0.10, the CIs shrank considerably (test: 1.10,0.85; independent: 1.10,0.90), but the confidence values dropped to 87.61% and 77.61%. An α of 0.01 achieved the optimal balance between interval tightness and coverage, providing 95.41% confidence with mean CI lengths of 1.70 for test and 1.80 for independent test set. Notably, this interval length is tighter than the standard 95% CI length of 1.96 for a Gaussian distribution, indicating improved efficiency.

**TABLE 2:** Uncertainty measurements (jackknife+) of MRGCN with RF model on test and independent test sets.

*α*	Confidence value (%) (*n* = 3515)	Test data		Independent data (Ind.)		Interval lengths	
		Original (%)	Predicted (%)	Original (%)	Predicted (%)	Test (mean ± std)	Ind. (mean ± std)
0.001	97.41	99.60	100	98.98	100	3.30 ± 0.13	3.40 ± 0.15
0.005	96.61	98.13	100	89.80	100	2.10 ± 0.12	2.10 ± 0.16
0.01	95.61	96.12	99.87	86.73	100	1.70 ± 0.13	1.80 ± 0.17
0.05	87.61	84.89	98.40	74.49	98.98	1.10 ± 0.14	1.10 ± 0.19
0.10	77.61	75.94	96.66	70.41	96.94	0.85 ± 0.15	0.90 ± 0.19

*Note:* α: independent parameter; Confidence Value: Confidence values calculated based on the α values; Original and Predicted: Percentage of true and predicted $\log P_{\mathrm{app}}$ values within the corresponding confidence intervals; Interval Lengths: Mean of the predicted confidence interval length ± standard deviation.

With this configuration, 96.12% of the true $\log P_{\mathrm{app}}$ values in the test set and 86.73% in the independent test set fell within the predicted intervals. For the predicted $\log P_{\mathrm{app}}$ values, 99.87% of the test points and 100% of the independent test points lay inside the intervals. The results from Table S21 and Figure S3 indicated that the MRGCN representation exhibited a consistently higher Spearman rank correlation between the root mean square error (RMSE) and root mean variance (RMV) values across different regressors compared to other representations, indicating a stronger monotonic relationship between the predictive uncertainty and the prediction error. Subsequently, it can be concluded that larger prediction intervals corresponded to larger errors, supporting favorable uncertainty behavior and thereby confirming the robustness and efficiency of the best‐performing model.

### 3.2 Influence of Molecular Descriptors

**FIGURE 2:**
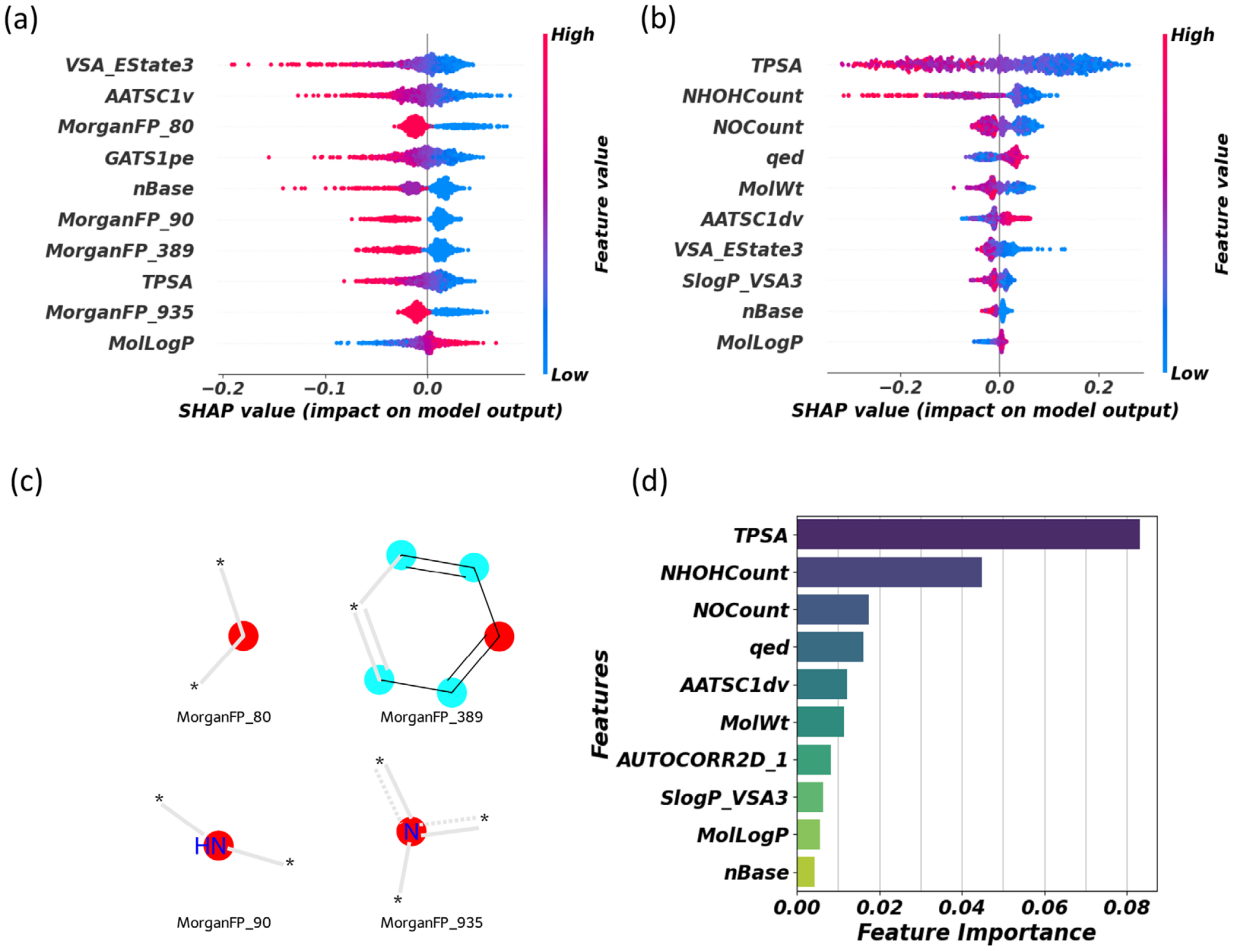
Most influencing features. (a) and (b) Top 10 most important features according to average absolute SHAP values for NN and RF model, respectively. Blue and red color indicated a low and high feature value. Each row in figures (a) and (b) represents the contribution summary of a single feature. For instance, in Figure (a) the last row illustrated the contribution of lipophilicity (MolLogP) of a compound in determining its permeability. The red points on the positive side of the vertical axis indicate that higher MolLogP values are associated with a positive contribution to permeability. (c) Most important Morgan fingerprints for NN model corresponding to those shown in Figure (a); (d) Top 10 features with the highest influence based on Gini index values for the RF model.

Figure 2a,b present the top 10 influential descriptors for NN and RF models, respectively, ranked by their average absolute SHAP values. The y‐axis lists descriptor names in descending order of importance, while the x‐axis shows their corresponding SHAP values. Feature values are color‐coded, with red indicating a higher value and blue indicating a lower value. For example, the descriptor nBase (number of basic groups in a molecule) from Figure 2a for the NN model indicated that high nBase values negatively affect the $\log P_{\mathrm{app}}$, while low nBase values corresponded to a positive effect. This is plausible as highly charged compounds can interfere with passive diffusion across the Caco‐2 cell membrane [27]. Similarly, a lower topological polar surface area $\left(\text{TPSA}\right)$ indicates a positive effect on the $\log P_{\mathrm{app}}$ value indicating a higher rate of permeation across the Caco‐2 membrane. This agrees with current literature as higher TPSA introduces stronger hydrogen bonding which can be unfavorable for drug penetration across the Caco‐2 membrane [28].

For the NN model, four substructures based on the Morgan fingerprints were found in the top‐10 most influential features as shown in Figure 2a. The substructures, obtained using the approach mentioned in Data S9, are presented in Figure 2c with the centered atom based on which the substructures of radius 2 were generated, marked in red and the aromaticity of an atom is denoted by cyan color. The SHAP analysis indicated that the four most influential substructures exhibited an inverse relationship with the $\log P_{\mathrm{app}}$ value. It further suggested that a molecule exhibiting high lipophilicity (MolLogP) was more likely to have a higher $\log P_{\mathrm{app}}$ value, consistent with the conclusion of previous studies [29].

For the RF model, TPSA, number of total N−H and O−H bonds $\left(\text{NHOHCount}\right)$; which indirectly represent the total number of hydrogen bond donors in a molecule, total number of nitrogen and oxygen atoms $\left(\text{NOCount}\right)$, quantitative estimation of drug‐likeliness $\left(\mathrm{QED}\right)$ and molecular weight $\left(\text{MolWt}\right)$ emerged as the most influential features. Among the top 5 most influential features identified in Figure 2b, four (TPSA,NHOHCount,NOCount and MolWt) exhibited an inverse relationship with $\log P_{\mathrm{app}}$ values, while only QED demonstrated a directly proportional relationship. This indicates that a molecule with high TPSA, MolWt, NHOHCount, or NOCount have a tendency to exhibit a lower $\log P_{\mathrm{app}}$ value, as noted in previous studies [29]. A broad spread in the SHAP value distributions of *NHOHCount* and *TPSA* suggest that the contribution of these features on the $\log P_{\mathrm{app}}$ value varies based on other features of the compound. Conversely, a limited spread in features *NOCount, MolWt, MolLogP* and *nBase* imply a sample‐independent effect on the $\log P_{\mathrm{app}}$ value. The feature importance calculated using Gini‐index [25] also established a similar set of important features with the same hierarchy as shown in Figure 2d.

**FIGURE 3:**
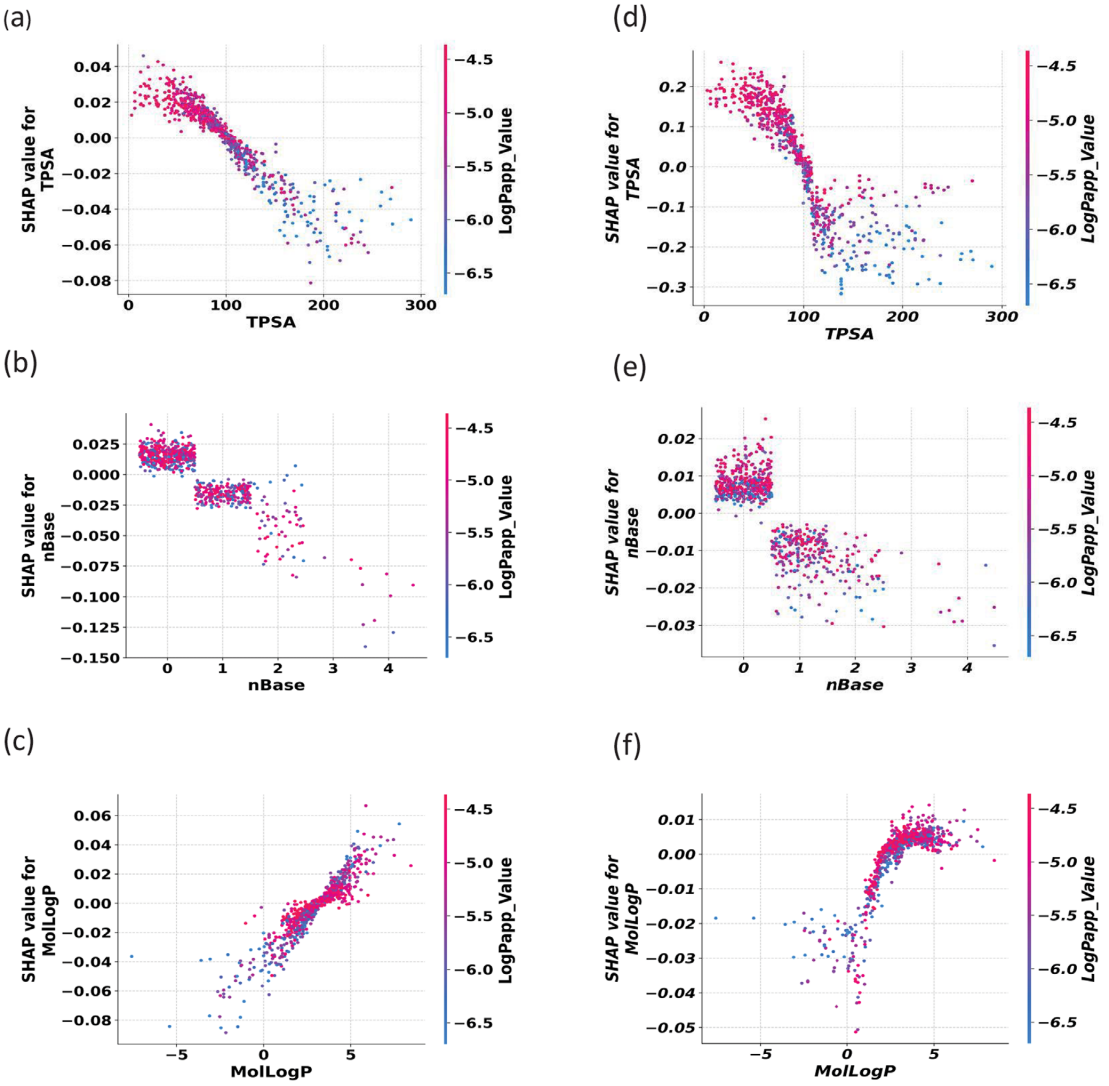
Dependence plots for the common most influential features for Neural Network (NN) and Random Forest (RF) models: (a, d) Topological polar surface area (TPSA); (b, e) Number of basic groups (nBase); (c, f) octanol–water partition coefficient (MolLogP). Subplots (a, b, c) and (d, e f) correspond to NN and RF models, respectively. The dependence plot portrays the relationship between a feature's value (x‐axis) and its contribution to the prediction (y‐axis). Each point represents an individual data sample. In another term, dependence plot illustrates that whether increasing or decreasing a feature value resulted in a consistent increased or decreased effect. As depicted by the color bar, the red and blue colors indicate datapoints associated with high and low log‐permeability values, respectively.

Furthermore, the SHAP dependence plot for three key features (*TPSA*, *nBase* and *MolLogP*) overlapped between NN and RF models were visualized to infer the feature importance in more detail. Figure 3 presents the plots with color coded datapoints according to the original $\log P_{\mathrm{app}}$ values. The x‐axis represents the feature values, and the y‐axis represents their corresponding SHAP values. Figure 3a,d indicated that *TPSA* values correlated negatively with SHAP values. The color gradient further suggests that the molecules with lower TPSA tended to have a higher $\log P_{\mathrm{app}}$ value. This trend was also observed in the SHAP values which decreased as TPSA increased.

The NN model exhibited a directly proportional relationship for *MolLogP*. In contrast, the RF model demonstrated a non‐linear relationship where a moderate increment in lipophilicity resulted in increased permeability, while the SHAP value dropped after a *MolLogP* value of 5.0 indicating a negative effect of lipophilicity on the permeability value. Such behaviors aligned with well‐established pharmacokinetic principles and the predictions of $P_{\mathrm{app}}$ agree with the Lipinski's rule of 5 [30], especially for the RF model.

**FIGURE 4:**
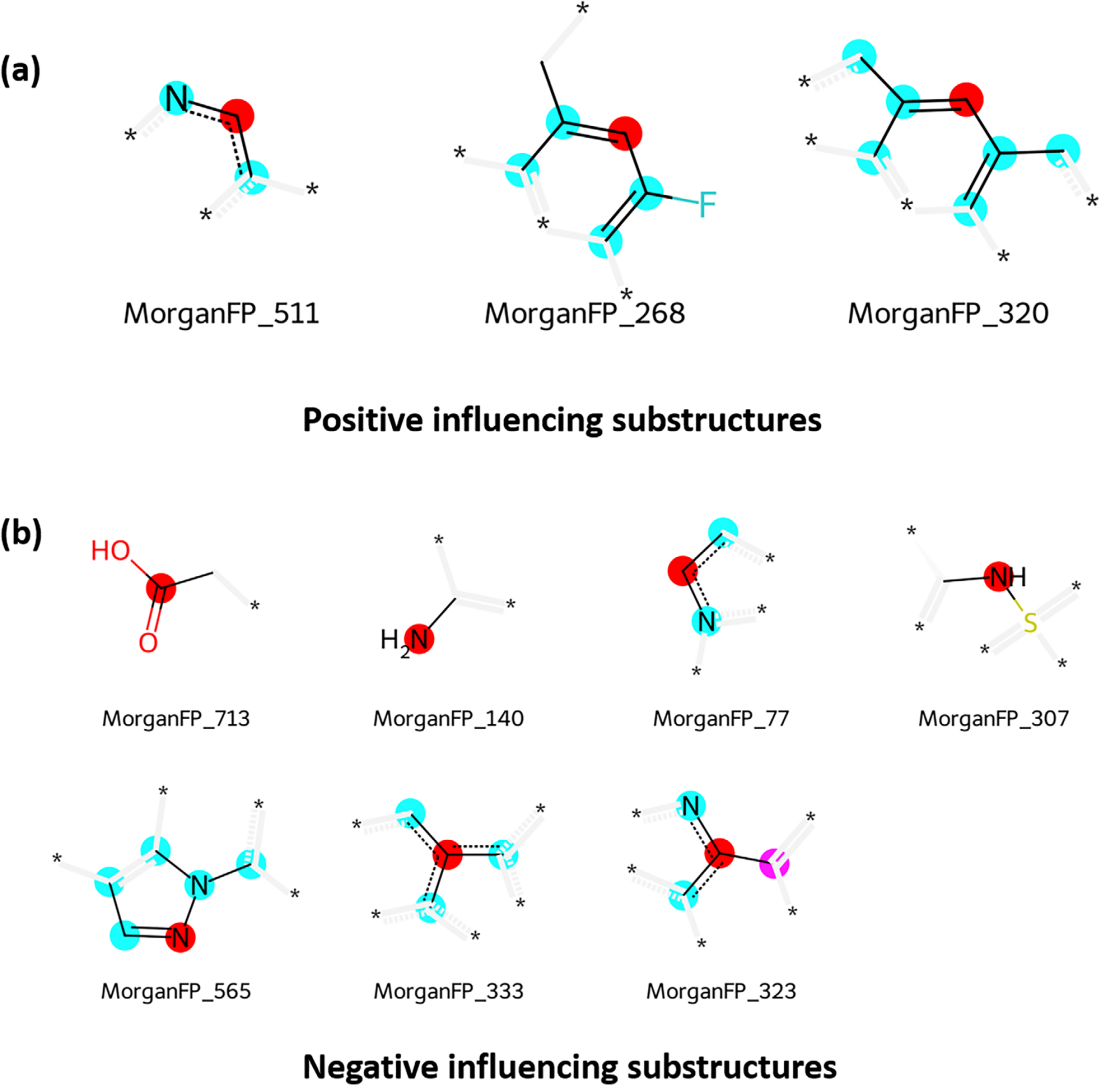
Common substructures (present in at least 50% of the test set) exhibiting (a) positive and (b) negative influences on the $\log P_{\mathrm{app}}$ value using SHAP analysis for the descriptors with RF model. These substructures were derived using Morgan fingerprints. In the figures, the red color denoted the central atom, cyan indicates aromatic atom, and the pink indicates the atom is a member of a non‐aromatic ring. The asterisks (*) indicates the attachment points where the substructure connects to the rest of the molecule.

Common substructures that influenced the $\log P_{\mathrm{app}}$ values either positively or negatively were assessed. However, there was not a single substructure found to appear in all compounds of the test set. Therefore, a more refined analysis was conducted to identify the common substructures in at least 50% of the test instances. Figure 4a,b illustrate the predominant substructures that positively and negatively influence the $\log P_{\mathrm{app}}$ prediction, respectively.

Overall, the SHAP value analysis justified the model (descriptors with RF and NN) capability to capture the physiochemical principles in terms of the predominant role of hydrogen donating capability, polarity, lipophilicity, and number of basic atoms in a molecule.

## 4 Discussion

The MRGCN‐based representation combined with RF regressor demonstrated the most consistent performance with suitable uncertainty‐aware characteristics across both the datasets among all the molecular feature representation techniques investigated, as evidenced from Tables 1, S8, S9, S21, and Figure S3. These observations highlighted that MRGCN‐based representations were effective at capturing intricate, rich, and generalizable information about molecules than molecular descriptor–based representations. Accordingly, the MRGCN with RF model was designated as the “best model”. Although only a marginal improvement was observed over the molecular descriptor–based representations for the independent test partition, its overall consistency and favorable uncertainty behavior supported this designation.

The Mol2Vec representation also achieved competitive predictive performance on the test set, benefiting from its learned chemical intuition through the unsupervised pre‐training process. In contrast, the VAE representation performed the worst among all the representations. This is likely because the VAE is trained solely on structural information without incorporating additional physicochemical descriptors (e.g., molecular weight, atomic weight, valency etc.) and thus captured only the underlying grammar of the SMILES strings. Consequently, the learned embeddings were less informative, reducing their effectiveness to predict $\log P_{\mathrm{app}}$ values. This highlights the importance of incorporating physicochemical knowledge alongside structural features to improve predictive performance.

### 4.1 Comparison With Previous Models

Table 3 presents the performance of the models in comparison with existing approaches across three evaluation metrics ($R^{2}$, RMSE, and MAE). The dataset sources by Falcón‐Cano et al. used in this study was considered as the baseline [4]. Among all the representation‐regressor combinations, molecular descriptors with the ϵ‐SVR model outperformed the baseline, yielding an $R^{2}$ value of 0.68. In addition, it achieved improved error metrics (RMSE = 0.42, MAE = 0.32), reflecting better predictive accuracy than the existing methods reported in Table 3. The MRGCN with RF model also surpassed both the baseline and the Wang et al. model [10], achieving an $R^{2}$ value of 0.65, the lowest MAE of 0.31, and a competitive RMSE of 0.43. The presented results indicated that both the ϵ‐SVR with molecular descriptors and the MRGCN with RF model delivered better performance compared to existing models, with the latter demonstrating better improvement in error robustness.

**TABLE 3:** Comparison with existing models.

Method	R^2^	RMSE	MAE
Falcón‐Cano et al. [4]	0.61	0.43	0.33
Wang et al. [4, 10]	0.55	—	0.39
𝛜‐SVR + Feature selection	**0.68**	**0.42**	0.32
MRGCN + RF	**0.65**	0.43	**0.31**

*Note:* Results outperforming the baseline model (Falcón et al.) are highlighted in bold font. $R^{2}$—Coefficient of determination, RMSE—root mean squared error & MAE—mean absolute error value between the original and predicted $\log P_{\mathrm{app}}$ value.

### 4.2 Limitations of the Model

The presented model demonstrates an improvement in performance compared to those previously presented, but it is important to acknowledge the limitations. An in‐depth error analysis was evaluated on the worst performing 10% test data to understand the reason for the predictive error. Each of the test molecules that performed the worst were compared with their nearest neighbor in the train set using the MRGCN representation. The nearest molecule in the train set was obtained using the k‐nearest neighbors (KNN) algorithm for each of the test molecules. The cases demonstrated in Figures S4–S7 indicated two special types of discrepancies in the less performing molecules. In Figure S4, both the train and test molecules contained the same set of atoms with similar overall orientations, but with subtle differences in the attaching position of a particular substructure to the main molecule. For example, in Figure S4 (b) the carboxyl group (−COOH) is attached to the para position with respect to the carbon bonded to sulfur (S) atom for the test molecule, but the same carboxyl group is attached to the ortho position with respect to the same carbon atom in the train molecule. Despite the structural similarity of these atoms, their $\log P_{\mathrm{app}}$ value differs by approximately 10‐fold. In the other case (Figure S5), the test and train molecules are identical except for a single atom or functional group. For example, in Figure S5 (a) the test and train molecules differ by a single atom (the −O− is replaced by −S−), but their $\log P_{\mathrm{app}}$ value differs by more than 10‐fold. Similarly, in Figure S5(b) the test and train molecules differ by a single fluorine atom (−F). This subtle modification also leads to a substantial change in the log $\log P_{\mathrm{app}}$ value, (i.e., from −5.09 to −6.52).

These findings suggest that similarity‐based models have some fundamental limitations. It is evident from the comparison between the predicted $\log P_{\mathrm{app}}$ value for the test data and the original $\log P_{\mathrm{app}}$ value for the corresponding train data in Figures S4 and S5 that the regression model overprioritizes the structural similarity by considering the closest training sample as a strong predictive anchor. As a result, the model fails to capture significant but subtle changes in a molecule. Such structurally similar pairs with significantly different bioactivities are called activity cliffs [11, 31]. Differentiating between these activity cliffs, which often correspond to near‐isomorphic graph structures, posed additional challenges for graph‐based molecular representations. As the activity cliffs have high structural similarity, analogous issues may also arise for other models that rely on computed descriptors, tokenizations, or similar representations for the same reason [32, 33, 34, 35].

Overall, the performance of structure‐based models dropped if the target value of a test molecule deviated substantially from that of structurally similar training molecules. Consequently, alternative and more expressive feature representation techniques are required that are capable of drawing a clear boundary between such activity cliffs. Graph isomorphic network [36], designed to differentiate isomorphic graphs based on local neighborhood structures, might be a good alternative to address such challenges and is therefore suggested as a promising direction for future research.

## 5 Conclusion

This study presented an in‐depth effectiveness analysis of four different numerical representations of a chemical compound from SMILES representation. The MRGCN based representation was found to be the better model in capturing generalized information about a compound among all the investigated representation methods, while the molecular descriptors‐based representation also exhibited good predictive performance. Model interpretation through SHAP values enhanced the reliability of learned models by revealing that learning was consistent with well‐established physiochemical principles. Furthermore, the uncertainty analysis through the jackknife+ technique predicted confidence intervals and to a certain extent described the robustness of the final model. Although there are limitations associated with this model for compounds with similar atomic composition having closely related isomorphic graph structures, the developed ML models offer improved accuracy in Caco‐2 apparent permeability prediction compared to those published previously.

## Author Contributions

All authors wrote the manuscript. A.O., R.R., and S.S. designed the research. R.M., R.R., and S.S. performed the research, analyzed the data and models.

## Funding

This work has been internally funded by the University of Liverpool.

## Conflicts of Interest

A.O. is Director and CSO for Extentus Pharma Ltd. and is co‐inventor of patents relating to long‐acting injectable medicines. A.O. has been co‐investigator on funding received by the University of Liverpool or Extentus Pharma Ltd. from ViiV Healthcare, Bicycle Therapeutics and Gilead Sciences and has received personal fees from Gilead, Shionogi and Assembly Biosciences. All other authors declared no competing interests for this work.

## Supporting information


**Data S1:** Supporting Information.

**Figure S1:** Common datapoints between each pair of source datasets. (a) Dataset 1 vs. Dataset 2; (b) Dataset 1 vs. Dataset3; (c) Dataset 2 vs. Dataset 3.
**Table S1:** Hyperparameters for MRGCN model.
**Table S2:** Variational auto‐encoder architecture. FS: Filter size, KS: Kernel size, FC: Fully‐connected layers, Nμσ: Sampling from Gaussian distribution.
**Table S3:** Hyperparameters for VAE model.
**Figure S2:** PCA, tSNE, and UMAP plots for all the molecular representations. Rows correspond to the different molecular representations and columns correspond to the dimensionality reduction methods. (a, b, c) Molecular descriptors; (d, e, f) MRGCN; (g, h, i) Mol2Vec; (j, k, l) VAE. (a, d, g, j) PCA; (b, e, h, k) t‐SNE; (c, f, i, l) UMAP. Gray, red, green, and blue color correspond to train, test, validation, and independent datapoints, respectively.
**Table S4:** Neural network hyperparameters for different representations.
**Table S5:** Random forest hyperparameters for different representations. #: Number of.
**Table S6:** Support vector machine hyperparameters for different representations.
**Table S7:** Potential set of hyperparameter values explored in the manual tuning process for the deep learning models.
**Table S8:** Ten‐fold cross‐validation results for the test and independent data, respectively.
**Table S9:** Bemis‐Murcko scaffold‐based split results for test and independent data, respectively.
**Table S10:** Jackknife+ results for MRGCN + SVM.
**Table S11:** Jackknife+ results for MRGCN + NN.
**Table S12:** Jackknife+ results for Descriptors + ϵ‐SVR.
**Table S13:** Jackknife+ results for Descriptors + RF.
**Table S14:** Jackknife+ results for Descriptors + NN.
**Table S15:** Jackknife+ results for Mol2Vec + ϵ‐SVR.
**Table S16:** Jackknife+ results for Mol2Vec + RF.
**Table S17:** Jackknife+ results for Mol2Vec + NN.
**Table S18:** Jackknife+ results for VAE + ϵ‐SVR.
**Table S19:** Jackknife+ results for VAE + RF.
**Table S20:** Jackknife+ results for VAE + NN.
**Table S21:** Spearman rank correlation coefficients between RMSE and RMV values of the 10‐fold cross‐validation results for all the representation regressor combinations.
**Figure S3:** Root mean square error (RMSE) vs. root mean variance (RMV) plots for all representation regressor combinations. Rows correspond to the different molecular representations and columns correspond to different regressor models. (a, b, c) Molecular descriptors; (d, e, f) MRGCN; (g, h, i) Mol2Vec; (j, k, l) VAE. (a, d, g, j) RF; (b, e, h, k) ϵ‐SVR; (c, f, i, l) NN.
**Figure S4:** Representative examples of molecular activity cliffs in which test and nearest train set compounds share similar substructures but differ slightly in their spatial orientations.
**Figure S5:** Representative examples of molecular activity cliffs in which test and nearest train set compounds share the same substructures with a slight difference of atomic composition.
**Figure S6:** Nearest neighbors of the weak performing molecules using MRGCN embeddings.
**Figure S7:** Nearest neighbors of the weak performing molecules using MRGCN embeddings.
