# Supplementary material for: Reliable Apparent Permeability Prediction Through Benchmarking, Stability, Uncertainty Quantification, and Explainable Machine Learning
Source: CPT Pharmacometrics Syst Pharmacol. 2026 Aug 13;15(8):e70305. doi: 10.1002/psp4.70305 (PMC13471647; doi:10.1002/psp4.70305)
Supplement: Supplementary file 1 [file PSP4-15-e70305-s002.docx]

**Supplementary Information for Reliable Apparent Permeability Prediction through Benchmarking, Stability, Uncertainty Quantification, and Explainable Machine Learning**

**Rajdeep Mondal^1,2^, Rajith KR Rajoli^3,4^, Andrew Owen^3,4^, Soumitra Samanta^1^**

^1^ Ramakrishna Mission Vivekananda Educational and Research Institute, Belur, India.

^2^ Institute for Advancing Intelligence, TCG CREST, Kolkata, India.
^3^ Centre of Excellence for Long-Acting Therapeutics, University of Liverpool, Liverpool, UK.

^4^ Department of Pharmacology and Therapeutics, University of Liverpool, Liverpool, UK.

## S1. Data collection and Pre-processing

The Caco-2 permeability dataset was constructed from three publicly available sources [1, 2, 3], as referenced in [4]. Each dataset contained experimentally measured Caco-2 permeability values for a diverse set of compounds with their corresponding SMILES representation [5]. The dataset from Wang et al.  contained 1272 data points [1], while the dataset published by Wang and Chen consisted of 1827 data points [3]. In both cases, permeability values were reported on the ${log}_{10}(P_{app}\times{10}^{-6} cm/s)$ scale. A relatively larger dataset was subsequently published incorporating 4464 data points with values originally reported on $P_{app}\times{10}^{-6} cm/s$ [2]. For consistency, the entire dataset was converted to ${log}_{10}(P_{app}\times{10}^{-6} cm/s)$ scale. Permeability values of the common datapoints among the source datasets are plotted in Figure S1 to observe the agreement between the reported values.

**Figure S1:** Common datapoints between each pair of source datasets. (a) Dataset 1 vs Dataset 2; (b) Dataset 1 vs Dataset3; (c) Dataset 2 vs Dataset 3.

 **The dotted* green line indicates the $\pm0.5$ interval around the exact agreement line $y=x$. Dataset 1, Dataset 2, and Dataset 3 correspond to Wang et al. (2016) [1], Wang and Chen (2020) [2], and Wang et al. (2020) [3], respectively.

## S2. Feature representations

### S2.1. Molecular descriptor representations

Two-dimensional molecular descriptors for each compound were computed using RDKit [6] and Mordred [7] library. For example, molecular descriptors, including properties such as molecular weight, total polar surface area (TPSA), quantitative estimate of drug-likeness (QED), number of hydrogen bond donors and acceptors, etc., were computed to construct the feature vector. Specifically, a total of $1613$ Mordred 2D descriptors (e.g., Wiener index, MOE-type descriptors, BalabanJ index), $1024$-bit morgan fingerprints (radius $2$), and $405$ RDKit-based physicochemical descriptors (e.g., molecular weight, LogP, TPSA, QED) were calculated and considered as the initial feature set. After removing non-numeric descriptors, the feature set retained $895$ Mordred descriptors, $390$ RDKit descriptors, and $1024$ morgan fingerprints, resulting in a total of $2309$ descriptors for each compound. Such descriptor-based representations captured physicochemical and structural properties at the molecular level.

A series of feature selection steps were employed to enhance the quality of the feature set and eliminate non-informative or redundant features: (i) features with zero variance were excluded; (ii) among pair of features with a Pearson correlation coefficient (PCC) $\geq0.99$, only one was retained; (iii) features with a PCC $\leq0.001$ with respect to the target variable (i.e., $logP_{app}$ values) were removed; (iv) features with mutual information $\leq0.01$ with the target variable were also discarded.

The final refined set retained $642$ molecular descriptors ($220$ from RDKit, $237$ from mordred, and $185$ from morgan fingerprints) after applying the selection criteria (as listed below). These features were normalized and used as the classical molecular descriptor representation for the prediction of apparent permeability.

["TPSA", "NHOHCount", "NOCount", "qed", "NumHeteroatoms", "fr_NH1", "NdO", "AUTOCORR2D_1", "nBondsD", "MolWt", "ATS0Z", "EState_VSA10", "AUTOCORR2D_57", "AUTOCORR2D_62", "SlogP_VSA1", "SlogP_VSA3", "Kappa3", "AUTOCORR2D_64", "AUTOCORR2D_61", "PEOE_VSA1", "AUTOCORR2D_94", "SMR_VSA10", "NumHAcceptors", "AUTOCORR2D_95", "NssNH", "nO", "AUTOCORR2D_60", "AUTOCORR2D_93", "AUTOCORR2D_96", "IC3", "IC2", "ATSC1pe", "BertzCT", "NumRotatableBonds", "SlogP_VSA2", "AUTOCORR2D_58", "VSA_EState3", "PEOE_VSA2", "EState_VSA1", "ATS0dv", "AUTOCORR2D_59", "AUTOCORR2D_92", "AUTOCORR2D_48", "EState_VSA2", "Xc-3d", "GGI4", "nN", "AUTOCORR2D_47", "SdssC", "ATSC0Z", "ZMIC2", "PEOE_VSA12", "AUTOCORR2D_46", "ZMIC1", "VSA_EState2", "IC1", "AUTOCORR2D_71", "GGI6", "ZMIC3", "AUTOCORR2D_91", "MorganFP_80", "GGI5", "nBondsKD", "HallKierAlpha", "AUTOCORR2D_89", "ATSC1v", "NdssC", "AUTOCORR2D_45", "Xpc-4d", "AUTOCORR2D_42", "Xc-3dv", "nBase", "MATS1pe", "AUTOCORR2D_87", "SMR_VSA1", "AATSC1pe", "Xpc-4dv", "MATS1dv", "ATSC0se", "NdNH", "AATSC1dv", "AUTOCORR2D_86", "AUTOCORR2D_70", "NddssS", "TSRW10", "nS", "fr_amide", "AUTOCORR2D_44", "AUTOCORR2D_69", "fr_sulfonamd", "fr_C_O", "AUTOCORR2D_43", "AUTOCORR2D_53", "SMR_VSA2", "AUTOCORR2D_41", "GATS2p", "GATS2i", "SsssCH", "NsssCH", "CIC0", "AATSC1v", "NsOH", "AUTOCORR2D_85", "AUTOCORR2D_49", "MIC2", "PEOE_VSA10", "SMR_VSA4", "VSA_EState9", "AUTOCORR2D_68", "MIC1", "ATSC1se", "AUTOCORR2D_65", "MorganFP_379", "fragCpx", "MaxAbsEStateIndex", "AUTOCORR2D_56", "VSA_EState5", "MorganFP_350", "GATS1dv", "ATSC0i", "GATS2v", "MinEStateIndex", "AUTOCORR2D_83", "AUTOCORR2D_81", "MorganFP_551", "fr_Al_COO", "fr_amidine", "AUTOCORR2D_169", "fr_NH2", "MorganFP_128", "MorganFP_389", "JGI10", "MorganFP_452", "MorganFP_983", "AUTOCORR2D_52", "JGI1", "AUTOCORR2D_66", "AUTOCORR2D_54", "nAcid", "MolLogP", "MinAbsEStateIndex", "AUTOCORR2D_55", "C1SP2", "SMR_VSA3", "MorganFP_607", "MorganFP_42", "RotRatio", "AUTOCORR2D_51", "MorganFP_966", "AvgIpc", "AUTOCORR2D_145", "fr_guanido", "AUTOCORR2D_84", "IC0", "C2SP3", "AATS1i", "SMR_VSA5", "ATSC1Z", "GATS2se", "fr_N_O", "MorganFP_623", "AATSC1se", "Xc-5dv", "MorganFP_762", "AATS1se", "MIC0", "MorganFP_759", "AXp-1dv", "JGT10", "ATSC3Z", "fr_COO", "MorganFP_895", "Xc-5d", "AUTOCORR2D_101", "ATSC2d", "GATS1Z", "ATSC5v", "MorganFP_730", "ATSC5p", "MorganFP_335", "AATS1Z", "MorganFP_878", "MorganFP_992", "PEOE_VSA13", "NssCH2", "ATSC3v", "MATS1se", "fMF", "MorganFP_90", "AUTOCORR2D_105", "VSA_EState4", "GATS1pe", "AUTOCORR2D_153", "MorganFP_739", "AUTOCORR2D_97", "NumAliphaticHeterocycles", "MorganFP_998", "MorganFP_815", "MorganFP_79", "SlogP_VSA4", "AUTOCORR2D_177", "MorganFP_823", "MorganFP_147", "MorganFP_563", "MorganFP_41", "MorganFP_190", "MorganFP_10", "NumSaturatedHeterocycles", "ATSC7se", "MorganFP_289", "MorganFP_902", "MorganFP_689", "AATSC0p", "MorganFP_176", "SssS", "MorganFP_325", "AATSC2p", "ATSC2p", "MorganFP_307", "FpDensityMorgan3", "FpDensityMorgan2", "MorganFP_151", "MorganFP_492", "MorganFP_207", "AUTOCORR2D_141", "GATS1i", "MorganFP_140", "MorganFP_579", "MorganFP_692", "C1SP3", "AATSC0d", "MorganFP_5", "MorganFP_820", "PEOE_VSA3", "MorganFP_112", "AUTOCORR2D_104", "MorganFP_263", "MorganFP_231", "MorganFP_721", "AATSC2d", "AATSC2v", "AUTOCORR2D_129", "ATSC5Z", "MorganFP_897", "MorganFP_562", "C2SP2", "MATS2i", "Xc-4d", "MorganFP_611", "n6Ring", "MATS2se", "JGI9", "nG12Ring", "ATSC5i", "MorganFP_469", "BalabanJ", "ATSC3p", "MorganFP_1019", "MorganFP_53", "ATSC3dv", "RingCount", "NumAromaticCarbocycles", "AATS3se", "GATS1p", "AATSC2i", "ATSC5dv", "MorganFP_401", "MorganFP_1", "fr_ketone", "AATS3i", "ATSC2i", "ATSC1d", "MorganFP_832", "ATSC2v", "PEOE_VSA6", "ATSC2se", "MorganFP_486", "MorganFP_301", "AATSC0Z", "MorganFP_583", "SMR_VSA6", "PEOE_VSA9", "FractionCSP3", "GATS3v", "AUTOCORR2D_137", "MorganFP_352", "MorganFP_29", "AATSC1d", "MorganFP_691", "fr_Ar_NH", "AATS1dv", "MATS2Z", "MorganFP_972", "fr_Al_OH", "EState_VSA6", "FpDensityMorgan1", "AATSC1Z", "MorganFP_209", "C1SP1", "Xc-4dv", "GATS2Z", "MorganFP_102", "MorganFP_191", "AATS0se", "MorganFP_511", "MorganFP_641", "PEOE_VSA7", "SlogP_VSA6", "NsssN", "Xch-7dv", "SMR_VSA7", "MorganFP_267", "ATSC4d", "n6aRing", "ATSC5se", "SPS", "ATSC3se", "EState_VSA3", "MorganFP_493", "MorganFP_172", "SlogP_VSA5", "MorganFP_203", "NumSaturatedRings", "n6AHRing", "AUTOCORR2D_152", "ATSC7m", "MorganFP_981", "MorganFP_135", "AUTOCORR2D_120", "AATSC2Z", "MorganFP_852", "AUTOCORR2D_144", "MorganFP_480", "MorganFP_782", "MorganFP_184", "AUTOCORR2D_191", "AATSC1i", "MATS1Z", "AATSC0i", "MorganFP_761", "AATS0m", "AATSC3Z", "AATSC1p", "PEOE_VSA8", "NumAliphaticRings", "AUTOCORR2D_113", "AUTOCORR2D_134", "AATS0v", "MorganFP_525", "MorganFP_942", "AATSC3se", "MorganFP_505", "JGI7", "nFHRing", "ATSC4se", "fr_piperdine", "EState_VSA9", "AATS3v", "SaaO", "MorganFP_585", "MorganFP_116", "n11AHRing", "MorganFP_913", "AATS2Z", "C3SP3", "MorganFP_935", "n5AHRing", "SlogP_VSA12", "nFRing", "AUTOCORR2D_114", "VSA_EState7", "fr_lactone", "GATS2dv", "SsF", "MATS3Z", "AUTOCORR2D_178", "AUTOCORR2D_112", "MorganFP_843", "SssssC", "AUTOCORR2D_117", "MorganFP_904", "MorganFP_793", "MorganFP_385", "AUTOCORR2D_119", "nHRing", "VSA_EState1", "MorganFP_786", "Xc-6dv", "nX", "JGI4", "n10FaRing", "NaaN", "MorganFP_625", "MorganFP_926", "ATSC6i", "MorganFP_597", "AUTOCORR2D_131", "AATSC3dv", "AUTOCORR2D_161", "AUTOCORR2D_167", "AUTOCORR2D_135", "AUTOCORR2D_183", "AUTOCORR2D_110", "Xch-6dv", "MorganFP_800", "MATS3v", "MorganFP_740", "AUTOCORR2D_102", "Xch-7d", "AUTOCORR2D_162", "AATS0i", "MorganFP_407", "FilterItLogS", "NssssC", "AUTOCORR2D_103", "MorganFP_817", "AATS3p", "MorganFP_136", "SIC0", "MorganFP_930", "MorganFP_131", "ATSC1p", "MorganFP_667", "fr_alkyl_halide", "MorganFP_415", "ATSC4dv", "AATSC0se", "AATS3d", "SaasC", "MorganFP_383", "MorganFP_458", "MorganFP_875", "MorganFP_239", "ATSC7d", "MorganFP_699", "AUTOCORR2D_170", "MorganFP_656", "PEOE_VSA11", "MorganFP_464", "JGI3", "n6ARing", "MorganFP_432", "fr_aniline", "AUTOCORR2D_109", "MorganFP_117", "AUTOCORR2D_142", "AATSC0v", "NaasC", "MorganFP_83", "SlogP_VSA8", "EState_VSA5", "MorganFP_312", "AATS3dv", "MorganFP_76", "NumAromaticRings", "ECIndex", "MorganFP_722", "AUTOCORR2D_136", "ATSC6v", "MorganFP_73", "MorganFP_46", "AUTOCORR2D_118", "MorganFP_795", "AUTOCORR2D_189", "GATS3Z", "n6HRing", "EState_VSA8", "MorganFP_125", "MorganFP_436", "MorganFP_534", "AUTOCORR2D_168", "MorganFP_738", "GATS1se", "ATSC8d", "fr_pyridine", "MorganFP_980", "SMR_VSA9", "MorganFP_71", "ATSC7i", "JGI2", "AUTOCORR2D_106", "AUTOCORR2D_133", "AUTOCORR2D_138", "MorganFP_749", "nFaRing", "MorganFP_216", "ATSC6m", "MorganFP_758", "AUTOCORR2D_160", "nG12FARing", "AUTOCORR2D_151", "MorganFP_896", "StsC", "AUTOCORR2D_154", "AUTOCORR2D_111", "VSA_EState6", "GATS3p", "AUTOCORR2D_182", "MorganFP_610", "MorganFP_406", "AUTOCORR2D_159", "AATSC3p", "ATSC4v", "AATS3Z", "MorganFP_409", "fr_allylic_oxid", "MorganFP_961", "MorganFP_59", "NdsCH", "NsCH3", "MorganFP_649", "AATSC2se", "Ipc", "MorganFP_283", "SaaaC", "MorganFP_445", "MorganFP_130", "MorganFP_338", "fr_ether", "NumAliphaticCarbocycles", "MorganFP_962", "MorganFP_333", "Xch-4d", "AUTOCORR2D_107", "AUTOCORR2D_149", "AATSC0dv", "AUTOCORR2D_150", "ATSC8v", "MorganFP_523", "nG12FRing", "MorganFP_873", "MorganFP_596", "AUTOCORR2D_187", "AUTOCORR2D_100", "MorganFP_456", "AUTOCORR2D_116", "ATSC2Z", "NaaaC", "MorganFP_816", "ATSC8dv", "MorganFP_31", "AUTOCORR2D_164", "MorganFP_619", "ATSC8se", "AUTOCORR2D_186", "MorganFP_264", "MATS3se", "ATSC4i", "AUTOCORR2D_98", "AUTOCORR2D_165", "fr_imidazole", "MorganFP_633", "AUTOCORR2D_192", "n8FARing", "AUTOCORR2D_157", "MorganFP_694", "MorganFP_967", "PEOE_VSA5", "GATS3dv", "NumAromaticHeterocycles", "ATSC8Z", "Xch-6d", "nBridgehead", "MorganFP_680", "MorganFP_842", "AATS0d", "MorganFP_509", "MorganFP_165", "MorganFP_808", "JGI8", "fr_Ar_N", "MorganFP_552", "MorganFP_602", "AUTOCORR2D_158", "MorganFP_504", "MorganFP_544", "WPath", "ATSC6se", "MorganFP_69", "SlogP_VSA11", "AUTOCORR2D_166", "SlogP_VSA7", "JGI5", "AUTOCORR2D_139", "n5Ring", "SssCH2", "MorganFP_911", "ATSC4Z", "AUTOCORR2D_163", "MorganFP_36", "PEOE_VSA4", "PEOE_VSA14", "AUTOCORR2D_181", "MorganFP_337", "AATS2d", "n5aRing", "n4Ring", "MorganFP_378", "MATS3i", "ATSC8i", "SIC3", "AUTOCORR2D_143", "MorganFP_858", "MorganFP_38", "AUTOCORR2D_147", "MorganFP_121", "Xc-6d", "AUTOCORR2D_185", "AUTOCORR2D_188", "ATSC3i", "MorganFP_20", "AUTOCORR2D_180", "AUTOCORR2D_155", "ATSC6dv", "Xch-3dv", "MorganFP_138", "MorganFP_886", "SlogP_VSA10", "TopoShapeIndex", "MorganFP_54", "MorganFP_829", "SdsN", "fr_NH0", "AUTOCORR2D_140", "SRW05", "AUTOCORR2D_148", "MorganFP_985", "MorganFP_361", "MorganFP_110", "n6aHRing", "ATSC4p", "MorganFP_855", "MorganFP_1017", "ATSC1i", "C3SP2", "MorganFP_885", "AUTOCORR2D_179", "MorganFP_346", "MorganFP_173", "MorganFP_219", "AATS0p", "MorganFP_1004"]

### S2.2. Multi-relational graph convolution network (MRGCN) representation

The MRGCN is a specialized variant of MRGCN designed to handle graphs with multiple types of relations between the nodes [8]. This method captures not only the atomic-level structural information but also the relative interactions between atoms within a chemical compound. A three-dimensional adjacency tensor $A\in R^{r\times n\times n}$, defined as $A_{ijk}=1$, if the $i^{th}$ bond type present between atoms with indices $j$ and $k$ and $A_{ijk}=0$ otherwise, is used to encode the presence of $r$ bond types among $n$ atoms in a compound. Then, the degree tensor $D\in R^{r\times n\times n}$ is computed as follows: $D_{ijj}=\sum_{k} A_{ijk}$ and $D_{ijk}=0 \forall j\neq k$. Each slice $A_{i},D_{i}\in R^{n\times n}$ corresponds to the adjacency and degree matrices for the $i^{th}$ relation, respectively.

The initial node features contained $20$ atom-level descriptors for each atom in a compound. Of these, $15$ descriptors were derived using RDKit, including both numerical features (e.g., partial charges, atomic weight, valency) and categorical features (e.g., hybridization state, chirality, aromaticity). In addition, atomic level log P contribution values were also included in the feature set, leveraging the data from Cheng et al. [9]. To further enhance the feature set, four more atomic descriptors (van der Waals radius, Sanderson electronegativity, polarizability, and Pauling electronegativity) were incorporated based on values from Castillo et al. [10] and WebElements [11]. Categorical features were one-hot encoded to convert them into a numerical format.

Different descriptor combinations were evaluated on the validation set to select the most informative features. Interestingly, the best-performing configuration contained $17$ descriptors, all of which were numerical; none of the categorical features were included. This suggested that the structural information encoded in the adjacency and degree tensors might have already captured the information conveyed by categorical features. The optimal combination of node-level descriptor matrix was denoted as $H^{(0)}\in R^{n\times17}$, where each row represents a 17-dimensional feature vector for an atom. Using these initial features, along with the adjacency and degree tensors, a $7$-layered MRGCN model was trained to obtain refined node embeddings. The layer-wise propagation rule was defined as:

$$H^{(l+1)}=\sigma\left( \sum_{i=1}^{r} D_{i}^{-\frac{1}{2}}\hat{A}_{i}D_{i}^{-\frac{1}{2}}H^{(l)}W_{i}^{(l)} \right)$$

Where, (i) $\hat{A}_{i}=A_{i}+I$, with $I$ as the identity matrix (self-connections added); (ii) $H^{(l)}$ denoted the node feature matrix at the $l^{th}$ layer; (iii) $W_{i}^{(l)}$ represented the learnable weight matrix for the $i^{th}$ relation at the $l^{th}$ layer; (iv) $\sigma(\cdot)$ was the activation function (e.g., rectified linear unit (ReLU)).

The dimensionality of the node feature vectors was empirically set to $500$, and the dimensions of $W_{i}^{(l)}$ were adjusted accordingly. Finally, to obtain a fixed-size representation for each molecule, average pooling was applied over all atoms within a graph.

**Table S1:** Hyperparameters for MRGCN model

| **Hyperparameters for MRGCN** | | | | |
| --- | --- | --- | --- | --- |
| **Learning Rate** | **Dropout** | **Epochs** | **Layers*** | **Activation function** |
| 0.001 | 20% | 500 | (17), 97, 178, 258, 339, 419, (500) | ReLU |

*The hidden layers were computed by dividing the representation’s input dimension (17) $-$ output dimension (500) into the corresponding number of hidden layers. The input (green colored) and output dimension (red colored) are provided in the table in the brackets.

### S2.3. Variational auto-encoder (VAE) representation

The VAE is a deep generative model that learns a continuous latent space using an encoder–decoder framework. All compounds were characterized by continuous latent vectors learned using the variational auto-encoder (VAE)  [12] framework. The SMILES were first represented in an one-hot encoded form using a vocabulary of size 63 with tokens: ["BH3-", "BH2-", "Ca+2", "NH+", "BH-", "CH-", "cH-", "Na+", "Cl-", "Br-", "SeH", "nH", "N+", "O-", "Br", "Cl", "n+", "N-", "P+", "Si", "S+", "PH", "C+", "C-", "B-", "Sn", "c-", "CH", "o+", "SH", "s+", "Se", "K+", "=", "N", "O", "1", "(", "C", ")", "c", "n", "3", "2", "S", "#", "o", "[", "]", "4", "s", "-", "F", "5", "P", "6", "B", "I", "7", "8", "9", "p", "."]. These tokens were derived by not only splitting the smiles tokens into individual symbols but rather partitioning into smaller units based on the design rule of the SMILES as given in the literature [5].

Each SMILES string was encoded as a matrix of dimension $250 \times63$, as the longest SMILES in the pre-training dataset contained 250 tokens. The encoder takes the SMILES $(x)$ as the input in form of a one-hot matrix and projects it into a lower-dimensional latent vector $(z)$, and the decoder tries to reconstruct the input $(x)$ from its corresponding latent vector. In this process, the encoder learns an approximate posterior distribution $q(z|x)$ over the latent vectors $(z)$ conditioned on a given SMILES $(x)$, whereas the decoder learns the conditional likelihood $p(x\mid z)$ over the SMILES $(x)$ which models the probability of reconstructing the original SMILES sequence from the latent representation. The encoder and decoder are jointly trained to achieve a posterior distribution $q(z|x)$ such that it remains close to the standard normal distribution, while simultaneously minimizing the reconstruction error between the original and decoded input.

The VAE architecture used to derive the feature representation was similar to the network designed by Samanta et al. [13]. Molecules were represented using a low-dimensional continuous feature set derived from VAE [12, 13]. The encoder constituted three 1-D convolutional layers with ReLU activation functions, followed by a fully connected layer with scale exponential linear unit (SELU) activation, and an additional fully connected layer to produce the latent representation. The decoder contained a fully connected layer with SELU activation, followed by four GRU blocks (each with a hidden size of $500$), and a linear layer using a softmax activation function. The latent dimension was empirically set to $300$, and the resulting $300$-dimensional latent vector served as a continuous molecular representation.

**Table S2:** Variational auto-encoder architecture. FS: Filter size, KS: Kernel size, FC: Fully-connected layers, $\mathcal{N}\left( \mu,\sigma\right)$: Sampling from Gaussian distribution

| **VAE Architecture** | | |
| --- | --- | --- |
| **Encoder (Input:** $\boldsymbol{x}\boldsymbol{\in}\boldsymbol{R}^{\boldsymbol{250\times63}}$**)** | **Reparameterization (**$\boldsymbol{\mu}\boldsymbol{,}\boldsymbol{\sigma}$**)** | **Decoder (Input:** $\boldsymbol{z}$**)** |
| 1. 1D Conv. FS=9, KS = 9, ReLU 2. 1D Conv. FS=9, KS = 9, ReLU 3. 1D Conv. FS=10, KS = 11, ReLU 4. Flatten 5. FC layer. Output dim = 300, SeLU 6. FC layer. Output dim = 300, FC layer. Output dim = 300   **Output =** $\boldsymbol{\mu,\sigma}\boldsymbol{\in}\boldsymbol{R}^{\boldsymbol{300}}$ | $\boldsymbol{z}\boldsymbol{\sim}\mathcal{N}\left( \boldsymbol{\mu}\boldsymbol{,diag(exp(}\boldsymbol{\sigma} \right)\boldsymbol{))}$  **Output:** $\boldsymbol{z \in}\boldsymbol{R}^{\boldsymbol{300}}$ | 1. FC layer, Output dim = 300, SeLU. 2. 4 GRU, Hidden dim = 500. 3. Z reshaped 4. Softmax   **Output:** $\tilde{\boldsymbol{x}}\boldsymbol{\in}\boldsymbol{R}^{\boldsymbol{250 \times63}}$ |

**Table S3:** Hyperparameters for VAE model

| **Hyperparameters for VAE** | |
| --- | --- |
| **Learning Rate** | **Dropout** |
| 0.0001 | 10% |

### S2.4. Mol2Vec representation

The Mol2Vec [14] model is a natural language processing (NLP) based deep learning model and used to represent molecules as fixed-dimensional continuous vectors. In this approach, for each atom in a molecule, substructures of radius 0 and 1 are generated using Morgan fingerprints. These substructures are then ordered sequentially according to the atom sequence in the SMILES string, forming a “chemical sentence". The Word2Vec algorithm is then applied to a large corpus of molecules to learn vector embeddings for each substructure [15]. Finally, the molecular representation is obtained by aggregating the embeddings of all substructures present in the molecule. Use of predefined characters in SMILES representations allows researchers to draw insightful relationships between the chemical representations and natural language.

### S2.5 Visualization of molecular embeddings in 2D using PCA, t-SNE and UMAP

Widely popular lower-dimensional embedding techniques, namely, principal component analysis (PCA), t-Distributed Stochastic Neighbor Embedding (t-SNE), and Uniform Manifold Approximation and Projection (UMAP) were used to visualize the learned representation and to examine their relative distributional structure across different data partitions.
**Figure S2:** PCA, tSNE, and UMAP plots for all the molecular representations. Rows correspond to the different molecular representations and columns correspond to the dimensionality reduction methods. (a, b, c) Molecular descriptors; (d, e, f) MRGCN; (g, h, i) Mol2Vec; (j, k, l) VAE. (a, d, g, j) PCA; (b, e, h, k) t-SNE; (c, f, i, l) UMAP. Gray, red, green, and blue colour correspond to train, test, validation, and independent datapoints, respectively.

## S3. Regression models

### S3.1. Neural networks

Fully connected feedforward neural networks (NNs) were also used in this study as regressors with varying numbers of linear layers depending on the dimension of the feature representations. Neural networks are popular deep learning models composed of input, hidden, and output layers along with various non-linear activation functions to increase model complexity. Several linear layers, specifically 7, 5, 10 and 5 were used for Molecular descriptors, MRGCN, VAE and Mol2Vec-based molecular representations, respectively. Each hidden layer was followed by a ReLU activation function, layer normalization, and dropouts. The mean squared error (MSE) loss was minimized using the adaptive moment estimation (Adam) optimizer to train the model. Optimal hyperparameters configuration (*i.e.,* learning rate, dropout percentage, and number of training epochs) were selected based on the validation set performance as summarized in Table S4. Notably, the VAE representation required a deeper network compared to MRGCN and Mol2Vec, likely because it captured only global molecular structures but failed to capture the local structural information that additionally incorporated into the graph and Mol2Vec-based representations.

**Table S4:** Neural network hyperparameters for different representations.

| **Feature**  **Representations** | **NN Hyperparameters** | | | |
| --- | --- | --- | --- | --- |
|  | **Molecular  descriptors** | **MRGCN** | **VAE** | **Mol2Vec** |
| Learning rate | 0.0001 | 0.0005 | 0.0003 | 0.0003 |
| Dropout | 20% | 20% | 30% | 30% |
| Epochs | 500 | 500 | 500 | 500 |
| No. of hidden layers | 7 | 5 | 10 | 5 |
| Activation func. | ReLU | ReLU | ReLU | ReLU |
| Hidden layers* | (642), 561, 481,  401, 321, 241, 161, 81, (1) | (500), 416, 333,  250, 167, 84, (1) | (300), 272, 245,  218,191, 164,  136, 109, 82, 55, 28, (1) | (300), 250, 200, 150, 100, 50, (1) |

*These hidden layers were computed by dividing the representation’s input dimension $-$ output dimension (1) into the corresponding number of hidden layers. The input (green colored) and output dimension (red colored) are provided in the table in the brackets.

### S3.2. Random forest

Random forest is an ensemble learning method that builds multiple decision trees for regression, and the final prediction is obtained by averaging the outputs of individual trees [16]. Hyperparameters of the RF model, including the number of decision trees, minimum number of samples in a leaf node, number of features considered for splitting at an internal node, and the choice of splitting criterion, were optimized. Based on validation performance, the optimal configuration was achieved with 500 decision trees, the minimum number of samples in a leaf node set to $2$, the number of features set to the square root of the total number of features for splitting at an internal node, and squared error as the splitting criterion.

**Table S5:** Random forest hyperparameters for different representations. #: Number of.

| **Feature**  **Representations** | **RF Hyperparameters** | | | |
| --- | --- | --- | --- | --- |
|  | **Molecular  descriptors** | **MRGCN** | **VAE** | **Mol2Vec** |
| # Decision trees | 500 | 500 | 500 | 500 |
| # Min leaf samples | 2 | 3 | 2 | 2 |
| # Splitting features | Sqrt | Sqrt | Sqrt | Sqrt |
| Splitting criterion | Squared error | Squared error | Squared error | Squared error |

### S3.3. Epsilon-support vector regressor

The $\epsilon$-SVR is a kernel-based method that extends support vector machines (SVMs) to regression models [17]. It seeks a function that best captures the relationship between input features and the target variable. Hyperparameter tuning was performed for the $\epsilon$-SVR model, including the kernel function, kernel coefficient, polynomial degree (for the polynomial kernel), margin of tolerance, and regularization parameter to obtain the optimal configuration. Validation showed that the best performance was obtained using a RBF kernel, kernel coefficient $\gamma$^[1]^, a regularization parameter of $1.0$, and a margin of tolerance of $0.1$. This configuration was adopted for all $\epsilon$-SVR models in this study.

**Table S6:** Support vector machine hyperparameters for different representations

| **Feature**  **Representations** | **RF Hyperparameters** | | | |
| --- | --- | --- | --- | --- |
|  | **Molecular  descriptors** | **MRGCN** | **VAE** | **Mol2Vec** |
| Kernel function | RBF | RBF | RBF | RBF |
| Kernel coefficient | $\gamma$ | $\gamma$ | $\gamma$ | $\gamma$ |
| Margin of tolerance | 0.1 | 0.1 | 0.1 | 0.1 |
| Regularization parameter | 1.0 | 1.0 | 1.0 | 1.0 |

## S4. Hyperparameter selection strategy

Hyperparameters were selected using validation-set performance via grid search for random forest and support vector regression models, whereas for deep learning models (i.e. - MRGCN, NN, and VAE) manual tuning was performed and the configuration demonstrating least predictive error value on the validation set was considered as the optimal configuration. Manual tuning was performed with a relevant subset of candidate values listed in Table S7 for different models. The hidden dimensions were determined by evenly interpolating between the input and output dimensions across the specified number of hidden layers. For example, a network with input dimension of 300 and four hidden layers (denoted with box) has dimensions: $300\to\boxed{240\to180\to120\to60}\to1$

**Table S7:** Potential set of hyperparameter values explored in the manual tuning process for the deep learning models

| **Hyperparameters** | **Values** |
| --- | --- |
| Learning rate | 0.001, 0.003, 0.005, 0.0001, 0.0003, 0.0005 |
| Optimizer | RMSProp, Adam, AdamW |
| Number of hidden layers | 3, 4, …..., 10 |
| Activation functions | ReLU, SeLU, Tanh |
| Output dimensions | GCN: 200, 300, 500 |
|  | VAE: 100, 200, 300, 400. 500 |
| Dropout | 10%, 20%, 30%, 40% |

## S5. Evaluation Metrics

The $R^{2}$ value indicates how well the model explains the variance in the underlying data; higher $R^{2}$ values indicate better model fitting. RMSE measures the standard deviation of the squared differences between the true and predicted values. MAE, in contrast, calculates the average magnitude of the absolute differences between the true and predicted values. Consequently, lower RMSE and MAE values correspond to superior predictive performance.

For a reference value, $t$, a $x\%$ error margin represents the tolerance interval $[t-\frac{xt}{100},t+\frac{xt}{100}]$, within which a prediction is considered as correct, relative to the true value.

In Bland–Altman (BA) plots, the x-axis represents the mean of the observed and predicted measurements, and the y-axis represents their difference. The mean difference and standard deviation (SD) indicate the systematic bias and random variation, respectively. The interval $[mean-1.96\times SD, mean+1.96\times SD]$ defines the $95\%$ limits of agreement (shown as solid blue lines in Figures 1 (c) and 1 (d) in the manuscript).

## S6. Results for 10-fold cross-validations and Bemis-Murcko scaffold-based splitting

Two popular model evaluation strategies, namely, 10-fold cross validation and structure aware splitting, were further adopted to assess the reliability of the obtained results. The 10-fold cross validation was employed to verify whether the obtained performance resulted by any random chance.

In parallel, five distinct dataset partitions were generated from the underlying dataset using Bemis–Murcko (BM) scaffold split approach [18]. The BM split algorithm first identifies the core scaffolds of a molecule, usually including only the ring and their linker components in a molecule. The core scaffolds of the SMILES were derived using the ‘MurckoScaffold’ module from ‘rdkit.Chem.Scaffolds’. Then all the molecules were grouped together based on their canonical scaffold representation such that all molecules sharing an identical scaffold were assigned to the same group. Finally, the groups were shuffled randomly and partitioned into training, test, and validation sets approximately in 80:10:10 ratio, while ensuring that no scaffold was shared across different splits. The BM split algorithm ran for five times to get five distinct scaffold-based partitions.

The mean results for both the 10-fold cross validation and the scaffold-based splitting are reported in Table S8 and S9 with their corresponding standard deviations.

**Table S8:** Ten-fold cross-validation results for the test and independent data, respectively.

| **Representation** | **Regressor** | $\mathbf{R}^{\mathbf{2}}$ | **RMSE** | **MAE** | **10%** | **20%** |
| --- | --- | --- | --- | --- | --- | --- |
| **Test Dataset** | | | | | | |
| **Descriptors** | **SVM** | 0.72 $\pm$ 0.04 | 0.40 $\pm$ 0.03 | 0.30 $\pm$ 0.02 | 82.45 $\pm$ 2.36 | 99.15 $\pm$ 0.96 |
|  | **RF** | 0.69 $\pm$ 0.04 | 0.42 $\pm$ 0.03 | 0.33 $\pm$ 0.02 | 79.87 $\pm$ 3.66 | 98.86 $\pm$ 0.82 |
|  | **NN** | 0.70 $\pm$ 0.05 | 0.41 $\pm$ 0.03 | 0.30 $\pm$ 0.03 | 83.30 $\pm$4.27 | 98.37 $\pm$ 1.21 |
| **MRGCN** | **SVM** | 0.69 $\pm$ 0.04 | 0.41 $\pm$ 0.02 | 0.31 $\pm$ 0.02 | 82.36 $\pm$ 2.97 | 98.55 $\pm$ 0.87 |
|  | **RF** | 0.72 $\pm$ 0.04 | 0.39 $\pm$ 0.02 | 0.29 $\pm$ 0.02 | 83.88 $\pm$ 2.20 | 98.48 $\pm$ 0.75 |
|  | **NN** | 0.70 $\pm$ 0.04 | 0.41 $\pm$ 0.02 | 0.31 $\pm$ 0.02 | 82.79 $\pm$ 3.35 | 98.72 $\pm$ 0.68 |
| **Mol2Vec** | **SVM** | 0.59 $\pm$ 0.06 | 0.48 $\pm$ 0.02 | 0.37 $\pm$ 0.02 | 74.86 $\pm$ 3.23 | 98.55 $\pm$ 1.04 |
|  | **RF** | 0.62 $\pm$ 0.05 | 0.46 $\pm$ 0.03 | 0.36 $\pm$ 0.02 | 75.44 $\pm$ 4.31 | 97.69 $\pm$ 0.91 |
|  | **NN** | 0.66 $\pm$ 0.04 | 0.43 $\pm$ 0.03 | 0.32 $\pm$ 0.01 | 80.92 $\pm$ 3.47 | 97.94 $\pm$ 0.71 |
| **VAE** | **SVM** | 0.51 $\pm$ 0.07 | 0.52 $\pm$ 0.03 | 0.40 $\pm$ 0.02 | 71.33 $\pm$ 3.84 | 96.10 $\pm$ 1.83 |
|  | **RF** | 0.43 $\pm$ 0.06 | 0.56 $\pm$ 0.03 | 0.45 $\pm$ 0.03 | 63.60 $\pm$ 5.11 | 95.32 $\pm$ 2.02 |
|  | **NN** | 0.51 $\pm$ 0.08 | 0.52 $\pm$ 0.03 | 0.40 $\pm$ 0.04 | 70.67 $\pm$ 4.14 | 96.77 $\pm$ 1.35 |
| **Independent test data** | | | | | | |
| **Descriptors** | **SVM** | 0.52 $\pm$ 0.02 | 0.59 $\pm$ 0.01 | 0.40 $\pm$ 0.01 | 75.51 $\pm$ 1.36 | 90.62 $\pm$ 0.65 |
|  | **RF** | 0.57 $\pm$ 0.01 | 0.56 $\pm$ 0.01 | 0.39 $\pm$ 0.01 | 74.08 $\pm$ 0.86 | 92.86 $\pm$ 0.48 |
|  | **NN** | 0.51 $\pm$ 0.02 | 0.60 $\pm$ 0.01 | 0.40 $\pm$ 0.01 | 73.67 $\pm$ 2.62 | 92.04 $\pm$ 0.65 |
| **MRGCN** | **SVM** | 0.56 $\pm$ 0.02 | 0.57 $\pm$ 0.01 | 0.39 $\pm$ 0.01 | 74.08 $\pm$ 1.94 | 92.86 $\pm$ 1.36 |
|  | **RF** | 0.57 $\pm$ 0.03 | 0.56 $\pm$ 0.02 | 0.37 $\pm$ 0.01 | 76.53 $\pm$ 2.96 | 92.66 $\pm$ 1.05 |
|  | **NN** | 0.53 $\pm$ 0.03 | 0.59 $\pm$ 0.02 | 0.42 $\pm$ 0.02 | 73.37 $\pm$ 2.82 | 92.45 $\pm$ 1.10 |
| **Mol2Vec** | **SVM** | 0.40 $\pm$ 0.01 | 0.67 $\pm$ 0.01 | 0.50 $\pm$ 0.01 | 63.68 $\pm$ 1.46 | 89.49 $\pm$ 0.49 |
|  | **RF** | 0.52 $\pm$ 0.02 | 0.59 $\pm$ 0.01 | 0.42 $\pm$ 0.01 | 72.35 $\pm$ 1.12 | 91.94 $\pm$ 0.32 |
|  | **NN** | 0.49 $\pm$ 0.04 | 0.62 $\pm$ 0.02 | 0.42 $\pm$ 0.01 | 74.08 $\pm$ 1.87 | 91.02 $\pm$ 1.25 |
| **VAE** | **SVM** | 0.27 $\pm$ 0.01 | 0.73 $\pm$ 0.01 | 0.57 $\pm$ 0.01 | 53.77 $\pm$ 1.99 | 85.40 $\pm$ 0.84 |
|  | **RF** | 0.19 $\pm$ 0.01 | 0.77 $\pm$ 0.01 | 0.62 $\pm$ 0.01 | 47.76 $\pm$ 1.79 | 86.02 $\pm$ 0.69 |
|  | **NN** | 0.24 $\pm$ 0.02 | 0.75 $\pm$ 0.02 | 0.58 $\pm$ 0.02 | 53.26 $\pm$ 2.08 | 85.40 $\pm$ 1.18 |

**Table S9:** Bemis-Murcko scaffold-based split results for test and independent data, respectively.

| **Representation** | **Regressor** | $\mathbf{R}^{\mathbf{2}}$ | **RMSE** | **MAE** | **10%** | **20%** |
| --- | --- | --- | --- | --- | --- | --- |
| **Test Dataset** | | | | | | |
| **Descriptors** | **SVM** | 0.63 ± 0.02 | 0.46 ± 0.03 | 0.36 ± 0.02 | 76.59 ± 2.19 | 96.73 ± 1.34 |
|  | **RF** | 0.63 ± 0.01 | 0.46 ± 0.02 | 0.37 ± 0.02 | 74.79 ± 1.57 | 97.54 ± 1.00 |
|  | **NN** | 0.66 ± 0.03 | 0.44 ± 0.03 | 0.34 ± 0.01 | 77.95 ± 3.76 | 97.44 ± 1.13 |
| **MRGCN** | **SVM** | 0.66 ± 0.03 | 0.44 ± 0.03 | 0.34 ± 0.02 | 80.23 ± 2.03 | 97.29 ± 1.49 |
|  | **RF** | 0.69 ± 0.03 | 0.42 ± 0.03 | 0.32 ± 0.02 | 79.85 ± 2.21 | 97.39 ± 1.98 |
|  | **NN** | 0.66 ± 0.03 | 0.44 ± 0.02 | 0.34 ± 0.01 | 79.25 ± 1.95 | 97.05 ± 1.11 |
| **Mol2Vec** | **SVM** | 0.58 ± 0.03 | 0.49 ± 0.04 | 0.38 ± 0.03 | 73.82 ± 4.37 | 97.57 ± 1.09 |
|  | **RF** | 0.56 ± 0.03 | 0.50 ± 0.03 | 0.40 ± 0.03 | 69.94 ± 1.81 | 96.97± 1.51 |
|  | **NN** | 0.57 ± 0.04 | 0.49 ± 0.03 | 0.37 ± 0.02 | 74.21 ± 3.26 | 96.00 ± 1.49 |
| **VAE** | **SVM** | 0.49 ± 0.03 | 0.55 ± 0.04 | 0.42 ± 0.03 | 67.99 ± 2.62 | 95.36 ± 1.36 |
|  | **RF** | 0.39 ± 0.04 | 0.59 ± 0.03 | 0.48 ± 0.03 | 57.88 ± 3.25 | 94.02 ± 1.78 |
|  | **NN** | 0.42 ± 0.04 | 0.58 ± 0.03 | 0.45 ± 0.03 | 64.48 ± 5.17 | 93.89 ± 2.01 |
| **Independent test data** | | | | | | |
| **Descriptors** | **SVM** | 0.54 ± 0.02 | 0.58 ± 0.01 | 0.38 ± 0.02 | 76.94 ± 0.50 | 92.25 ± 0.50 |
|  | **RF** | 0.55 ± 0.02 | 0.57 ± 0.01 | 0.40 ± 0.01 | 72.45 ± 1.44 | 92.45 ± 0.50 |
|  | **NN** | 0.53 ± 0.03 | 0.58 ± 0.02 | 0.39 ± 0.01 | 74.29 ± 2.45 | 92.25 ± 0.82 |
| **MRGCN** | **SVM** | 0.55 ± 0.01 | 0.57 ± 0.02 | 0.39 ± 0.02 | 75.10 ± 1.22 | 92.66 ± 0.76 |
|  | **RF** | 0.56 ± 0.02 | 0.57 ± 0.01 | 0.38 ± 0.01 | 76.53 ± 1.12 | 92.25 ± 0.82 |
|  | **NN** | 0.52 ± 0.01 | 0.59 ± 0.01 | 0.42 ± 0.01 | 71.23 ± 1.35 | 92.86 ± 0.65 |
| **Mol2Vec** | **SVM** | 0.38 ± 0.01 | 0.67 ± 0.01 | 0.50 ± 0.01 | 63.27 ± 1.12 | 89.39 ± 0.50 |
|  | **RF** | 0.49 ± 0.01 | 0.61 ± 0.01 | 0.44 ± 0.01 | 69.39 ± 1.44 | 92.25 ± 0.82 |
|  | **NN** | 0.46 ± 0.04 | 0.63 ± 0.02 | 0.43 ± 0.03 | 71.23 ± 3.44 | 90.21 ± 1.78 |
| **VAE** | **SVM** | 0.28 ± 0.01 | 0.73 ± 0.01 | 0.57 ± 0.01 | 51.84 ± 1.98 | 88.37 ± 1.39 |
|  | **RF** | 0.18 ± 0.01 | 0.78 ± 0.01 | 0.62 ± 0.01 | 47.35 ± 1.53 | 86.73 ± 0.92 |
|  | **NN** | 0.25 ± 0.02 | 0.74 ± 0.01 | 0.58 ± 0.02 | 52.45 ± 2.86 | 88.17 ± 0.82 |

## S7. Jackknife+: uncertainty measurement of a model

The uncertainty involved in a model’s performance can be quantified by computing confidence intervals (CIs) for each datapoint. For non-linear models, conventional CI estimation techniques based on student’s $t$-distribution cannot be directly applied. Instead, another suitable model-agnostic method, jackknife+, was used to estimate the intervals.

The jackknife+ constructs predictive confidence intervals using $K$-fold cross-validation [19]. The CI with confidence parameter $\alpha$ for a new data point $X_{n+1}$ is given by:

$$C_{n,K,\alpha}(X_{n+1})=\left[ \hat{q}_{n,\alpha}^{-}\{\hat{\mu}_{-S_{k(i)}}(X_{n+1})-R_{i}^{CV}\}, \hat{q}_{n,\alpha}^{+}\{\hat{\mu}_{-S_{k(i)}}(X_{n+1})+R_{i}^{CV}\} \right]$$

where $\hat{\mu}_{-S_{k}}$ denotes the model (MRGCN with RF) trained on the data excluding the $k^{th}$ fold $S_{k}$, and $k(i)$ is the fold containing the $i^{th}$ data point. The terms $\hat{q}_{n,\alpha}^{-}$, $\hat{q}_{n,\alpha}^{+}$, and $R_{i}^{CV}$ are defined as:

$\hat{q}_{n,\alpha}^{+}\{v_{i}\}=the \lceil(1-\alpha)(n+1)\rceil^{th} smallest element of v_{1},v_{2},\ldots,v_{n}$.

$\hat{q}_{n,\alpha}^{-}\{v_{i}\}=the \lfloor\alpha(n+1)\rfloor^{th} smallest element of v_{1},v_{2},\ldots,v_{n}$;

$$R_{i}^{CV}=|Y_{i}-\hat{\mu}_{-S_{k(i)}}(X_{i})|, i=1,\ldots,n.$$

where ⌈𝑥⌉ and ⌊𝑥⌋ represent the ceiling and floor functions respectively.

The combined training and validation data ($n=3515$) were utilized as the cross-validation set with $K=10$ fold to compute the CIs for all the representation-regressor combinations. For jackknife+, the effective confidence level is given by $1-2\alpha-\sqrt{2/n}$, where $n$ is the number of cross-validation points. Table 2, S10-S20 reports the confidence values and CI lengths for five different $\alpha$ values $(0.001, 0.005, 0.01, 0.05, and 0.10$) evaluated on both the test and independent test sets. For each $\alpha$, an assessment was conducted to determine whether the ground-truth and predicted $logP_{app}$ values of the test data were contained within the corresponding intervals, and the tightness of confidence intervals was analysed with the variation of the independent parameter ($\alpha$).

Jackknife+ results for all representation-regressor combinations except MRGCN + RF (mentioned in the main manuscript) are provided in the following tables (S10-S20):

**Table S10:** Jackknife+ results for MRGCN + SVM

| $\boldsymbol{\alpha}$ | **Confidence Value** | **Test Data** | | **Independent Data (Ind.)** | | **Interval length** | |
| --- | --- | --- | --- | --- | --- | --- | --- |
|  |  | **Original (%)** | **Predicted (%)** | **Original (%)** | **Predicted (%)** | **Test** | **Ind.** |
| 0.001 | 97.41 | 99.87 | 100.00 | 98.98 | 100.00 | 3.53 $\pm$ 0.09 | 3.56 $\pm$ 0.12 |
| 0.005 | 96.61 | 98.40 | 100.00 | 93.88 | 100.00 | 2.31 $\pm$ 0.09 | 2.44 $\pm$ 0.13 |
| 0.01 | 95.61 | 95.72 | 100.00 | 91.84 | 100.00 | 1.82 $\pm$ 0.08 | 1.88 $\pm$ 0.15 |
| 0.05 | 87.61 | 85.16 | 99.33 | 76.53 | 96.94 | 1.14 $\pm$ 0.07 | 1.21 $\pm$ 0.15 |
| 0.10 | 77.61 | 77.01 | 97.73 | 66.33 | 95.92 | 0.89 $\pm$ 0.11 | 0.94 $\pm$ 0.18 |

**Table S11:** Jackknife+ results for MRGCN + NN

| **𝛼** | **Confidence Value** | **Test Data** | | **Independent Data (Ind.)** | | **Interval length** | |
| --- | --- | --- | --- | --- | --- | --- | --- |
|  |  | **Original (%)** | **Predicted (%)** | **Original (%)** | **Predicted (%)** | **Test** | **Ind.** |
| 0.001 | 97.41 | 99.73 | 100.00 | 98.98 | 100.00 | 3.45 $\pm$ 0.07 | 3.47 $\pm$ 0.12 |
| 0.005 | 96.61 | 98.13 | 100.00 | 93.88 | 100.00 | 2.27 $\pm$ 0.09 | 2.33 $\pm$ 0.12 |
| 0.01 | 95.61 | 96.39 | 99.87 | 90.82 | 100.00 | 1.77 $\pm$ 0.11 | 1.85 $\pm$ 0.11 |
| 0.05 | 87.61 | 84.89 | 99.06 | 75.51 | 96.94 | 1.13 $\pm$ 0.12 | 1.21 $\pm$ 0.13 |
| 0.10 | 77.61 | 77.94 | 97.46 | 64.29 | 94.90 | 0.89 $\pm$ 0.14 | 0.93 $\pm$ 0.15 |

**Table S12:** Jackknife+ results for Descriptors + $\epsilon$-SVR

| **𝛼** | **Confidence Value** | **Test Data** | | **Independent Data (Ind.)** | | **Interval length** | |
| --- | --- | --- | --- | --- | --- | --- | --- |
|  |  | **Original (%)** | **Predicted (%)** | **Original (%)** | **Predicted (%)** | **Test** | **Ind.** |
| 0.001 | 97.41 | 99.87 | 100.00 | 98.98 | 100.00 | 3.41 $\pm$ 0.05 | 3.43 $\pm$ 0.09 |
| 0.005 | 96.61 | 98.40 | 100.00 | 91.84 | 100.00 | 2.19 $\pm$ 0.06 | 2.22 $\pm$ 0.08 |
| 0.01 | 95.61 | 94.52 | 100.00 | 82.65 | 100.00 | 1.72 $\pm$ 0.07 | 1.78 $\pm$ 0.10 |
| 0.05 | 87.61 | 81.42 | 99.87 | 68.37 | 100.00 | 1.09 $\pm$ 0.10 | 1.12 $\pm$ 0.11 |
| 0.10 | 77.61 | 70.86 | 99.60 | 58.16 | 100.00 | 0.83 $\pm$ 0.10 | 0.85 $\pm$ 0.13 |

**Table S13:** Jackknife+ results for Descriptors + RF

| **𝛼** | **Confidence Value** | **Test Data** | | **Independent Data (Ind.)** | | **Interval length** | |
| --- | --- | --- | --- | --- | --- | --- | --- |
|  |  | **Original (%)** | **Predicted (%)** | **Original (%)** | **Predicted (%)** | **Test** | **Ind.** |
| 0.001 | 97.41 | 100.00 | 100.00 | 98.98 | 100.00 | 3.33 $\pm$ 0.03 | 3.38 $\pm$ 0.05 |
| 0.005 | 96.61 | 98.26 | 100.00 | 91.84 | 100.00 | 2.21 $\pm$ 0.04 | 2.27 $\pm$ 0.04 |
| 0.01 | 95.61 | 95.99 | 100.00 | 84.69 | 100.00 | 1.77 $\pm$ 0.06 | 1.85 $\pm$ 0.08 |
| 0.05 | 87.61 | 83.69 | 100.00 | 73.47 | 100.00 | 1.21 $\pm$ 0.05 | 1.24 $\pm$ 0.09 |
| 0.10 | 77.61 | 75.67 | 100.00 | 65.31 | 100.00 | 0.97 $\pm$ 0.08 | 0.99 $\pm$ 0.13 |

**Table S14:** Jackknife+ results for Descriptors + NN

| **𝛼** | **Confidence Value** | **Test Data** | | **Independent Data (Ind.)** | | **Interval length** | |
| --- | --- | --- | --- | --- | --- | --- | --- |
|  |  | **Original (%)** | **Predicted (%)** | **Original (%)** | **Predicted (%)** | **Test** | **Ind.** |
| 0.001 | 97.41 | 99.73 | 100.00 | 98.98 | 100.00 | 3.19 $\pm$ 0.04 | 3.24 $\pm$ 0.02 |
| 0.005 | 96.61 | 98.13 | 100.00 | 88.78 | 100.00 | 2.06 $\pm$ 0.03 | 2.11 $\pm$ 0.06 |
| 0.01 | 95.61 | 93.72 | 100.00 | 83.67 | 100.00 | 1.63 $\pm$ 0.03 | 1.65 $\pm$ 0.07 |
| 0.05 | 87.61 | 81.95 | 99.73 | 70.41 | 100.00 | 1.06 $\pm$ 0.05 | 1.12 $\pm$ 0.08 |
| 0.10 | 77.61 | 72.86 | 99.06 | 59.18 | 100.00 | 0.83 $\pm$ 0.06 | 0.89 $\pm$ 0.08 |

**Table S15:** Jackknife+ results for Mol2Vec + $\epsilon$-SVR

| $\boldsymbol{\alpha}$ | **Confidence Value** | **Test Data** | | **Independent Data (Ind.)** | | **Interval length** | |
| --- | --- | --- | --- | --- | --- | --- | --- |
|  |  | **Original (%)** | **Predicted (%)** | **Original (%)** | **Predicted (%)** | **Test** | **Ind.** |
| 0.001 | 97.41 | 100.00 | 100.00 | 98.98 | 100.00 | 3.78 $\pm$ 0.09 | 3.86 $\pm$ 0.07 |
| 0.005 | 96.61 | 98.66 | 100.00 | 92.86 | 100.00 | 2.58 $\pm$ 0.12 | 2.63 $\pm$ 0.13 |
| 0.01 | 95.61 | 96.12 | 100.00 | 86.73 | 100.00 | 2.06 $\pm$ 0.09 | 2.15 $\pm$ 0.08 |
| 0.05 | 87.61 | 83.82 | 100.00 | 72.45 | 100.00 | 1.32 $\pm$ 0.10 | 1.36 $\pm$ 0.11 |
| 0.10 | 77.61 | 74.60 | 100.00 | 61.22 | 100.00 | 1.07 $\pm$ 0.11 | 1.18 $\pm$ 0.09 |

**Table S16:** Jackknife+ results for Mol2Vec + RF

| **𝛼** | **Confidence Value** | **Test Data** | | **Independent Data (Ind.)** | | **Interval length** | |
| --- | --- | --- | --- | --- | --- | --- | --- |
|  |  | **Original (%)** | **Predicted (%)** | **Original (%)** | **Predicted (%)** | **Test** | **Ind.** |
| 0.001 | 97.41 | 100.00 | 100.00 | 98.98 | 100.00 | 3.29 $\pm$ 0.03 | 3.35 $\pm$ 0.06 |
| 0.005 | 96.61 | 97.99 | 100.00 | 89.80 | 100.00 | 2.26 $\pm$ 0.05 | 2.37 $\pm$ 0.07 |
| 0.01 | 95.61 | 95.72 | 100.00 | 84.69 | 100.00 | 1.86 $\pm$ 0.06 | 1.92 $\pm$ 0.07 |
| 0.05 | 87.61 | 83.69 | 100.00 | 70.41 | 100.00 | 1.27 $\pm$ 0.08 | 1.34 $\pm$ 0.09 |
| 0.10 | 77.61 | 76.47 | 100.00 | 60.20 | 100.00 | 1.03 $\pm$ 0.09 | 1.08 $\pm$ 0.11 |

**Table S17:** Jackknife+ results for Mol2Vec + NN

| **𝛼** | **Confidence Value** | **Test Data** | | **Independent Data (Ind.)** | | **Interval length** | |
| --- | --- | --- | --- | --- | --- | --- | --- |
|  |  | **Original (%)** | **Predicted (%)** | **Original (%)** | **Predicted (%)** | **Test** | **Ind.** |
| 0.001 | 97.41 | 100.00 | 100.00 | 98.98 | 100.00 | 3.46 $\pm$ 0.08 | 3.53 $\pm$ 0.07 |
| 0.005 | 96.61 | 97.73 | 100.00 | 91.84 | 100.00 | 2.23 $\pm$ 0.09 | 2.29 $\pm$ 0.09 |
| 0.01 | 95.61 | 93.85 | 100.00 | 85.71 | 100.00 | 1.76 $\pm$ 0.08 | 1.79 $\pm$ 0.06 |
| 0.05 | 87.61 | 81.02 | 99.87 | 68.37 | 100.00 | 1.12 $\pm$ 0.07 | 1.15 $\pm$ 0.08 |
| 0.10 | 77.61 | 73.40 | 98.40 | 63.27 | 98.98 | 0.88 $\pm$ 0.13 | 0.94 $\pm$ 0.15 |

**Table S18:** Jackknife+ results for VAE + $\epsilon$-SVR

| **𝛼** | **Confidence Value** | **Test Data** | | **Independent Data (Ind.)** | | **Interval length** | |
| --- | --- | --- | --- | --- | --- | --- | --- |
|  |  | **Original (%)** | **Predicted (%)** | **Original (%)** | **Predicted (%)** | **Test** | **Ind.** |
| 0.001 | 97.41 | 99.87 | 100.00 | 98.98 | 100.00 | 3.70 $\pm$ 0.10 | 3.78 $\pm$ 0.13 |
| 0.005 | 96.61 | 96.66 | 100.00 | 87.76 | 100.00 | 2.58 $\pm$ 0.12 | 2.69 $\pm$ 0.13 |
| 0.01 | 95.61 | 94.39 | 100.00 | 84.69 | 100.00 | 2.08 $\pm$ 0.13 | 2.17 $\pm$ 0.11 |
| 0.05 | 87.61 | 83.56 | 100.00 | 66.33 | 100.00 | 1.36 $\pm$ 0.12 | 1.42 $\pm$ 0.15 |
| 0.10 | 77.61 | 72.06 | 99.87 | 55.10 | 100.00 | 1.07 $\pm$ 0.14 | 1.12 $\pm$ 0.12 |

**Table S19:** Jackknife+ results for VAE + RF

| **𝜶** | **Confidence Value** | **Test Data** | | **Independent Data (Ind.)** | | **Interval length** | |
| --- | --- | --- | --- | --- | --- | --- | --- |
|  |  | **Original (%)** | **Predicted (%)** | **Original (%)** | **Predicted (%)** | **Test** | **Ind.** |
| 0.001 | 97.41 | 99.73 | 100.00 | 98.98 | 100.00 | 3.69 ± 0.09 | 3.82 ± 0.11 |
| 0.005 | 96.61 | 96.52 | 100.00 | 90.82 | 100.00 | 2.57 ± 0.11 | 2.71 ± 0.13 |
| 0.01 | 95.61 | 94.52 | 100.00 | 83.67 | 100.00 | 2.12 ± 0.14 | 2.29 ± 0.16 |
| 0.05 | 87.61 | 83.42 | 100.00 | 71.43 | 100.00 | 1.45 ± 0.14 | 1.53 ± 0.16 |
| 0.10 | 77.61 | 73.26 | 100.00 | 58.16 | 100.00 | 1.21 ± 0.16 | 1.34 ± 0.17 |

**Table S20:** Jackknife+ results for VAE + NN

| $\boldsymbol{\alpha}$ | **Confidence Value** | **Test Data** | | **Independent Data (Ind.)** | | **Interval length** | |
| --- | --- | --- | --- | --- | --- | --- | --- |
|  |  | **Original (%)** | **Predicted (%)** | **Original (%)** | **Predicted (%)** | **Test** | **Ind.** |
| 0.001 | 97.41 | 99.73 | 100.00 | 98.98 | 100.00 | 3.63 $\pm$ 0.07 | 3.72 $\pm$ 0.08 |
| 0.005 | 96.61 | 97.33 | 100.00 | 91.84 | 100.00 | 2.60 $\pm$ 0.10 | 2.75 $\pm$ 0.12 |
| 0.01 | 95.61 | 94.39 | 100.00 | 80.61 | 100.00 | 2.08 $\pm$ 0.13 | 2.13 $\pm$ 0.17 |
| 0.05 | 87.61 | 83.56 | 100.00 | 66.33 | 100.00 | 1.39 $\pm$ 0.17 | 1.48 $\pm$ 0.19 |
| 0.10 | 77.61 | 74.60 | 100.00 | 55.10 | 100.00 | 1.11 $\pm$ 0.19 | 1.23 $\pm$ 0.21 |

## S8. Comparison between the root mean square error (RMSE) Root Mean Variance (RMV)

The relationship between prediction error and predictive uncertainty was analysed by considering the root mean squared error (RMSE) and root mean variance (RMV) at the level of individual test data points. The 10-fold cross-validation results were used to compute the RMSE and RMV for each test datapoints. Similar to *jackknife+* uncertainty estimation method, the 10-fold cross-validation experiments were performed on the combined training and validation datasets. For each test data point, predictions from all folds were collected to quantify both error and uncertainty. Let $\hat{y}_{i}^{k}$be the predicted values for *i^th^* test instance on the *k^th^* fold and let *y_i_* denote the corresponding ground-truth value. The RMSE and RMV for the *i*^th^ test sample are computed as:

RMSE vs RMV plot for all representation-regressor combinations are shown in Figure S3. We further assessed the monotonic relationship between RMSE and RMV by computing the Spearman rank correlation coefficient, as shown in Table S21. A positive correlation indicates that samples associated with higher predictive uncertainty tend to exhibit larger prediction errors. The graph-based MRGCN representations consistently achieved higher correlation values than other representations, indicating a stronger monotonic relationship between predictive uncertainty and prediction error, thereby suggesting its favourable uncertainty characteristics that larger prediction intervals corresponded to larger errors. Among the regressors, the NN model better captures the monotonic relationship as described by higher correlation values of 0.378, 0.495, 0.506, and 0.411 for descriptor-based, MRGCN, Mol2Vec, and VAE representations, respectively.

| Representation | Regressor | Spearman *ρ* |
| --- | --- | --- |
| Descriptors | SVM | 0.205 |
|  | RF | 0.215 |
|  | NN | 0.378 |
| MRGCN | SVM | 0.436 |
|  | RF | 0.478 |
|  | NN | 0.495 |
| Mol2Vec | SVM | 0.162 |
|  | RF | 0.236 |
|  | NN | 0.506 |
| VAE | SVM | 0.181 |
|  | RF | 0.217 |
|  | NN | 0.411 |

**Table S21:** Spearman rank correlation coefficients between RMSE and RMV values of the 10-fold cross-validation results for all the representation regressor combinations.

(a)	(b)	(c)

(d)	(e)	(f)

(g)	(h)	(i)

(j)	(k)	(l)

**Figure S3:** Root mean square error (RMSE) vs root mean variance (RMV) plots for all representation regressor combinations. Rows correspond to the different molecular representations and columns correspond to different regressor models. (a, b, c) Molecular descriptors; (d, e, f) MRGCN; (g, h, i) Mol2Vec; (j, k, l) VAE. (a, d, g, j) RF; (b, e, h, k) *ϵ*-SVR; (c, f, i, l) NN.

## S9: Morgan bit to substructure mapping

Hash collision between different molecular substructures makes it impossible to define an inverse mapping between a particular Morgan bit and a unique substructure.

To overcome this issue, the following strategy was adopted to find a unique substructure corresponding to a Morgan bit. First, the atom environments contributing to each activated bit was tracked using the bit information generated during fingerprint construction leveraging RDKit’s bitInfo. For each fingerprint, a representative substructure was selected by scanning through a predefined list of SMILES. The first atom environment that matched with the RDKit’s bitInfo was assigned as the corresponding representative substructure.

## S10. Nearest neighbours for worst performing test molecules

Nearest (Figure S4, S5) and five nearest (Figure S6, S7) train compounds of some of the worst performing test molecules were found using MRGCN embeddings. The resulting examples are illustrated in Figures S4–S7. Many cases of activity cliffs [20, 21] were found as challenging due to the misleading information added by structurally similar training compounds.

**Figure S4:** Representative examples of molecular activity cliffs in which test and nearest train set compounds share similar substructures but differ slightly in their spatial orientations.

**Figure S5:** Representative examples of molecular activity cliffs in which test and nearest train set compounds share the same substructures with a slight difference of atomic composition.

**Figure S6:** Nearest neighbors of the weak performing molecules using MRGCN embeddings

**Figure S7:** Nearest neighbors of the weak performing molecules using MRGCN embeddings

# References

[1] N.-N. Wang *et al.*, “ADME properties evaluation in drug discovery: Prediction of Caco-2 cell permeability using a combination of NSGA-II and boosting,” *Journal of chemical information and modeling*, vol. 56, no. 4, pp. 763–773, 2016.

[2] X. Wang, M. Liu, L. Zhang, Y. Wang, Y. Li, and T. Lu, “Optimizing pharmacokinetic property prediction based on integrated datasets and a deep learning approach,” *Journal of Chemical Information and Modeling*, vol. 60, no. 10, pp. 4603–4613, 2020.

[3] Y. Wang and X. Chen, “QSPR model for Caco-2 cell permeability prediction using a combination of HQPSO and dual-RBF neural network,” *RSC advances*, vol. 10, no. 70, pp. 42938–42952, 2020.

[4] G. Falcón-Cano, C. Molina, and M. Á. Cabrera-Pérez, “Reliable prediction of Caco-2 permeability by supervised recursive machine learning approaches,” *Pharmaceutics*, vol. 14, no. 10, 2022.

[5] D. Weininger, “SMILES, a chemical language and information system. 1. Introduction to methodology and encoding rules,” *Journal of Chemical Information and Computer Sciences*, vol. 28, no. 1, pp. 31–36, 1988.

[6] “RDKit: Open-source cheminformatics.” <https://www.rdkit.org>. doi: <https://doi.org/10.5281/zenodo.591637>.

[7] H. Moriwaki, Y.-S. Tian, N. Kawashita, and T. Takagi, “Mordred: A molecular descriptor calculator,” *Journal of Cheminformatics volume*, vol. 10, no. 4, 2018.

[8] Z. Huang, X. Li, Y. Ye, and M. K. Ng, “MR-GCN: Multi-relational graph convolutional networks based on generalized tensor product,” in *Proceedings of the twenty-ninth international joint conference on artificial intelligence (IJCAI-20)*, 2020, pp. 1258–1264.

[9] T. Cheng *et al.*, “Computation of octanol- water partition coefficients by guiding an additive model with knowledge,” *Journal of chemical information and modeling*, vol. 47, no. 6, pp. 2140–2148, 2007.

[10] J. A. Castillo-Garit, Y. Marrero-Ponce, F. Torrens, and R. García-Domènech, “Estimation of ADME properties in drug discovery: Predicting Caco-2 cell permeability using atom-based stochastic and non-stochastic linear indices.” *Journal of pharmaceutical sciences*, vol. 975, 2007.

[11] “WebElements.” <https://www.webelements.com/>.

[12] D. P. Kingma, “Auto-encoding variational bayes,” *arXiv preprint arXiv:1312.6114*, 2013.

[13] S. Samanta, S. Hagan, N. Swainston, T. J. Roberts, and D. B. Kell, “VAE-sim: A novel molecular similarity measure based on a variational autoencoder,” *Molecules*, vol. 25, no. 15, p. 3446, 2020.

[14] S. Jaeger, S. Fulle, and S. Turk, “Mol2vec: Unsupervised machine learning approach with chemical intuition,” *Journal of chemical information and modeling*, vol. 58, no. 1, pp. 27–35, 2018.

[15] T. Mikolov, “Efficient estimation of word representations in vector space,” *arXiv preprint arXiv:1301.3781*, vol. 3781, 2013.

[16] L. Breiman, “Random forests,” *Machine Learning*, vol. 45, no. 1, pp. 5–32, 2001.

[17] V. Vapnik, S. Golowich, and A. Smola, “Support vector method for function approximation, regression estimation and signal processing,” *Advances in neural information processing systems*, vol. 9, 1996.

[18] Guy W. Bemis and Mark A. Murcko, “The Properties of Known Drugs. 1. Molecular Frameworks”, *Journal of Medicinal Chemistry*, vol. 39, no. 15, p. 2887-2893, 1996. https://doi.org/10.1021/jm9602928.

[19] R. F. Barber, E. J. Candes, A. Ramdas, and R. J. Tibshirani, “Predictive inference with the jackknife+,” *The Annals of Statistics*, vol. 49, no. 1, pp. 486–507, 2021.

[20] Moritz Walter, Jens M. Borghardt, Lina Humbeck, Miha Skalic. “Multi-task ADME/PK Prediction at Industrial Scale: Leveraging Large and Diverse Experimental Datasets”. M*olecular Informatics,* 2024, 43, e202400079. <https://doi.org/10.1002/minf.202400079> .

[21] Derek van Tilborg, Alisa Alenicheva, and Francesca Grisoni. Exposing the limitations of molecular machine learning with activity cliffs*. J Chem Inf Model*, 62(23):5938–5951. <https://doi.org/10.1021/acs.jcim.2c01073>.

1. 𝛾=$\frac{1}{\text{number of features }\times\text{ variance of the features}}$
